# Assessing risk factors for malaria and schistosomiasis among children in Misungwi, Tanzania, an area of co-endemicity: A mixed methods study

**DOI:** 10.1371/journal.pgph.0002468

**Published:** 2023-11-22

**Authors:** Claudia Duguay, Jacklin F. Mosha, Eliud Lukole, Doris Mangalu, Charles Thickstun, Elizabeth Mallya, Tatu Aziz, Cindy Feng, Natacha Protopopoff, Franklin Mosha, Alphaxard Manjurano, Alison Krentel, Manisha A. Kulkarni

**Affiliations:** 1 School of Epidemiology and Public Health, University of Ottawa, Ottawa, Canada; 2 National Institute of Medical Research Tanzania, Mwanza Research Centre, Mwanza, Tanzania; 3 Kilimanjaro Christian Medical University College, Moshi, Tanzania; 4 Department of Community Health & Epidemiology, Dalhousie University, Halifax, Canada; 5 Faculty of Infectious and Tropical Diseases, Disease Control Department, London School of Hygiene and Tropical Medicine, London, United Kingdom; 6 Bruyère Research Institute, Ottawa, Ontario, Canada; Federal University of Mato Grosso do Sul, BRAZIL

## Abstract

Malaria and schistosomiasis are two major parasitic vector-borne diseases that are a particular threat to young children in Sub-Saharan Africa. In the present study, we investigated factors that are associated with malaria, schistosomiasis, and co-infection among school-aged children, using an explanatory sequential mixed-methods approach. A cross-sectional study was conducted in January 2022 in Misungwi, Tanzania, that sampled 1,122 children aged 5 to 14 years old for malaria and schistosomiasis infection. Mixed-effect logistic regression models were used to assess the association between infection prevalence or seroprevalence, and environmental determinants that create favorable conditions for vectors and parasites and social determinants that relate to disease exposure. Community mapping combined with direct field observations were conducted in August 2022 in three selected villages from the cross-sectional study to understand specific water use behaviors and to identify potential malaria mosquito larval breeding sites and freshwater snail habitat. The prevalence of malaria, seroprevalence of schistosomiasis, and co-infection in this study were 40.4%, 94.3%, and 38.1%, respectively. Individual-level factors emerged as the primary determinants driving the association with infection, with age (every one-year increase in age) and sex (boys vs girls) being statistically and positively associated with malaria, schistosomiasis, and co-infection (*P*<0.05 for all). Community maps identified many unimproved water sources in all three villages that were used by humans, cattle, or both. We found that children primarily fetched water, and that unprotected wells were dedicated for drinking water whereas ponds were dedicated for other domestic uses and cattle. Although not identified in the community maps, we found hand pumps in all three villages were not in use because of unpleasant taste and high cost. This study improves our understanding of individual, social and environmental factors that are associated with malaria, schistosomiasis, and co-infection, which can inform potential entry points for integrated disease prevention and control.

## Background

Eighty percent of the world’s population are at risk of one or more vector-borne diseases [1]. Malaria and schistosomiasis are two of the major parasitic vector-borne diseases that are a particular threat to young children in rural areas of sub-Saharan Africa (SSA) [1, 2]. This is due to a complex interplay between their exposures to malaria and schistosomiasis vectors and intermediate hosts (*Anopheles* mosquitoes, *Biomphalaria* and *Bulinus* snails), parasites (*Plasmodium*, *Schistosoma)*, biological susceptibility, and adaptive capabilities (e.g., access to preventive measures and treatment) [3–5].

Human malaria is caused by five different *Plasmodium* parasites, with *P*. *falciparum* being the predominant species in SSA [6]. The dominant *Anopheles (An*.*)* vector mosquitoes in Africa include *An*. *arabiensi*s, *An*. *gambiae* sensu stricto (s.s.), and *An*. *funestus*, all with their own unique ecological niches [7, 8]. *An*. *funestus* and *An*. *gambiae* s.s. share similar feeding and biting habits as they are both anthropophilic, endophagic, and bite from dusk till dawn [9, 10].

Several important tools to control and prevent malaria have been introduced in the past two decades, including long lasting insecticidal nets (LLIN), early diagnosis with rapid diagnostic tests, and treatment with artemisinin-based combination therapy [4], with LLINs being the main vector control tool for preventing malaria. Two billion insecticide treated nets, including LLINs, have been distributed between 2004–2020 with 65% of households in SSA now owning at least one LLIN [11]. Despite efforts to combat the disease, malaria remains a significant public health challenge in Tanzania, accounting for 4.1% total global malaria deaths, ranking third behind Nigeria and the Democratic Republic of Congo [12].

In SSA, there are two forms of schistosomiasis caused by two different *Schistosoma* species each with their respective snail intermediate host–*S*. *mansoni* that infects *Biomphalaria* snails and causes intestinal schistosomiasis, and *S*. *haematobium* that infects *Bulinus* snails and causes urogenital schistosomiasis [13, 14]. Like malaria, environmental and sociodemographic factors are important for the survival of the *Schistosoma* species, snail intermediate hosts, and the transmission of schistosomiasis [15].

The transmission cycle for schistosomiasis is influenced by human behavior, specifically the practice of open defecation, where an infected person passes parasitic eggs through their urine or feces into a freshwater source (i.e. lakes, ponds, streams) [13]. The eggs hatch and release miracidia that penetrate freshwater snails (*Bulinus* in seasonal bodies of water, and *Biomphalaria* in permanent bodies of water) where they develop and are released back into the water as an infective form of the parasite (cercariae) [13, 15, 16]. The cercariae can remain in the water searching for a secondary host for up to 72 hours [17]. Once the cercariae burrows through the skin of the secondary host, it migrates to the circulatory system where it develops and matures into its adult form; male and female adult worms mate and reside in the mesenteric veins of the bladder or intestine, where female *Schistosoma* can then again release their eggs through the urine and feces of the host to continue the transmission cycle [16, 17]. The lifespan of adult *Schistosoma* can survive approximately 3–6 years within their human host [15].

Prevention strategies for schistosomiasis have primarily focused on mass drug administration (MDA) campaigns that deliver Praziquantel tablets without prior diagnosis to at-risk populations in endemic areas, at a frequency that depends on the endemicity of the community to reduce worm burden [13, 18–20]. Praziquantel is effective on both *Schistosoma* species and not only treats schistosomiasis, but reduces transmission by decreasing the number of worms in the host [21, 22]. Although more resource intensive and logistically complex, snail control with molluscicides and provision of safe water, sanitation, and hygiene (WASH) have also been critical for schistosomiasis prevention and control [14, 17, 23, 24].

Schistosomiasis does not typically result in death but can lead to long-term health challenges and chronic morbidity. Tanzania has been reported to have moderate to high prevalence of schistosomiasis (irrespective of species) with considerable geographic variation, with studies reporting a prevalence of schistosomiasis between 6% and 53% in a single region of Tanzania [25–27].

Co-infection is likely to occur where malaria and schistosomiasis are co-endemic. This is especially likely in the Lake Victoria Zone in Tanzania where malaria and schistosomiasis are both endemic. Four studies between the years of 2010 and 2017 determined the prevalence of malaria and intestinal schistosomiasis co-infection as between 22.6% and 27.2% in the Lake Victoria Zone region [28–31]. Co-endemicity is likely due to the interaction between environmental and social factors contributing to the spread of both diseases, however, there is a limited body of evidence on the risk factors associated with malaria and schistosomiasis co-infection [28–30, 32–36]. Identifying factors associated with mono- and co-infection are crucial for effective prevention strategies. By moving away from a purely disease-centered approach and towards broader and integrated disease prevention strategies, we can optimize the use of limited resources to eliminate these diseases. The goal of this research is to understand population vulnerabilities, and identify socio-ecological factors associated with malaria, schistosomiasis, and co-infection among school-aged children in Misungwi, Lake Victoria Zone, Tanzania, using an explanatory sequential mixed-methods approach.

## Methods

### Study setting

**Fig 1:**
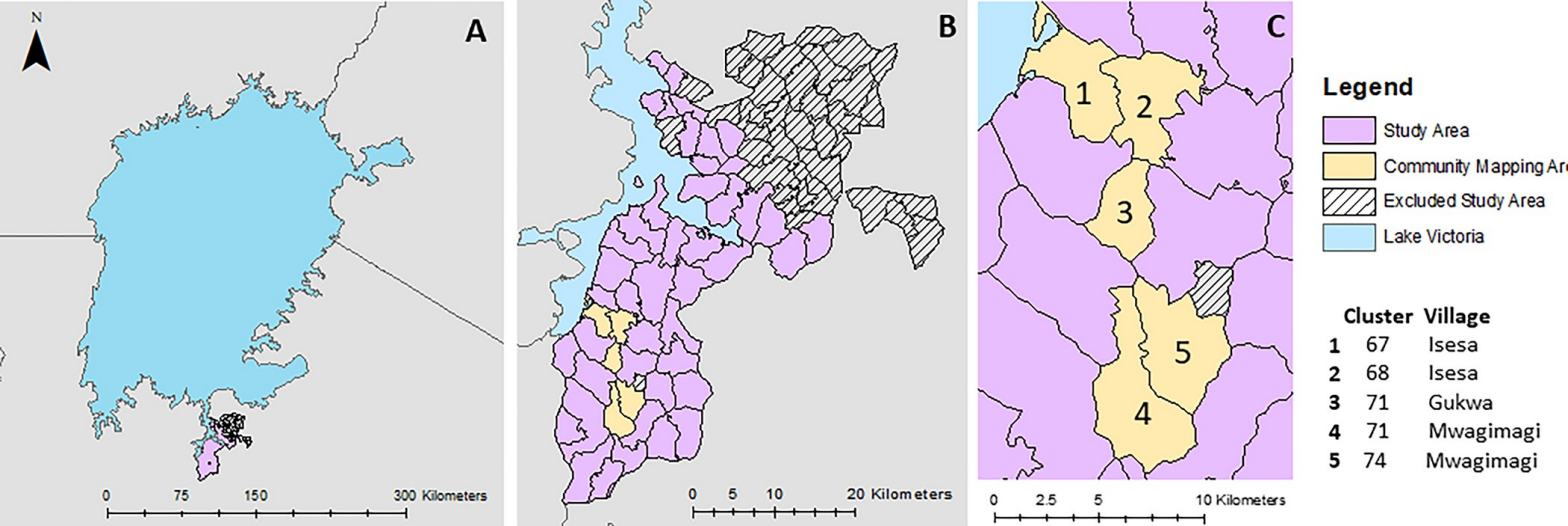
Map highlighting the study area in reference to Lake Victoria (A), a detailed view of the 48 study clusters (B), and the three villages selected for the community mapping component (C). Map content was produced with Esri ArcGIS software using study data and data provided by GADM and Natural Earth available online: https://gadm.org/download_country.html and https://www.naturalearthdata.com/.

This study was conducted in the district of Misungwi, which is located on the southern border of Lake Victoria in Tanzania (Fig 1A). Misungwi has two rainy seasons from March to May (Masika–long rains), and from October to January (Vuli–short rains). In the study region, *An*. *funestus s*.*l*. is the predominant malaria vector species, followed by *An*. *gambiae s*.*l*. [37]. Misungwi, and subsequently the Lake Victoria Zone, provides ample habitat for *Biomphalaria* snail species (intermediate host for intestinal schistosomiasis) that are present all year round in and around Lake Victoria, and for *Bulinus* snail species (intermediate host for urogenital schistosomiasis) found in seasonal water bodies and rice fields situated inland [25, 38, 39]. Misungwi covers an area of over 2100 km^2^ and includes 27 wards, 78 villages and a population of approximately 351,000 people; this area was divided into 86 clusters according to the main study protocol of a cluster randomized trial of malaria vector control interventions, 48 of which were used for this study (Fig 1B) [40].

### Sample size

The availability of limited funds necessitated schistosomiasis testing to be conducted on a smaller sample of children—1,296 children compared to 4,200. Although we collected survey information for all 4,200 children, the analysis was restricted to the subset of children that were tested for schistosomiasis (1,296 children). The sample size of 1,296 children (27 children per cluster in 48 clusters instead of 50 children per cluster in 84 clusters) was determined based on hypothesized schistosomiasis seroprevalence. Using the sample size equation for two proportions in a cross-sectional survey with clustered sampling design, this sample size was sufficient to estimate the seroprevalence of schistosomiasis (as measured by IgG and IgM antibody detection) with 80% power and 5% precision, assuming a seroprevalence of 50% and an intracluster correlation of 0.23 [26]. Under these assumptions it allowed us to detect an absolute 20% difference in anti-*Schistosoma* IgM and IgG antibody prevalence between two groups for any hypothesized risk factors (e.g., low and high risk) [41, 42]. A detailed explanation of the calculation can be found in the (S1 Text: Sample Size Derivation).

### Study design

#### Cross-sectional survey

**Fig 2:**
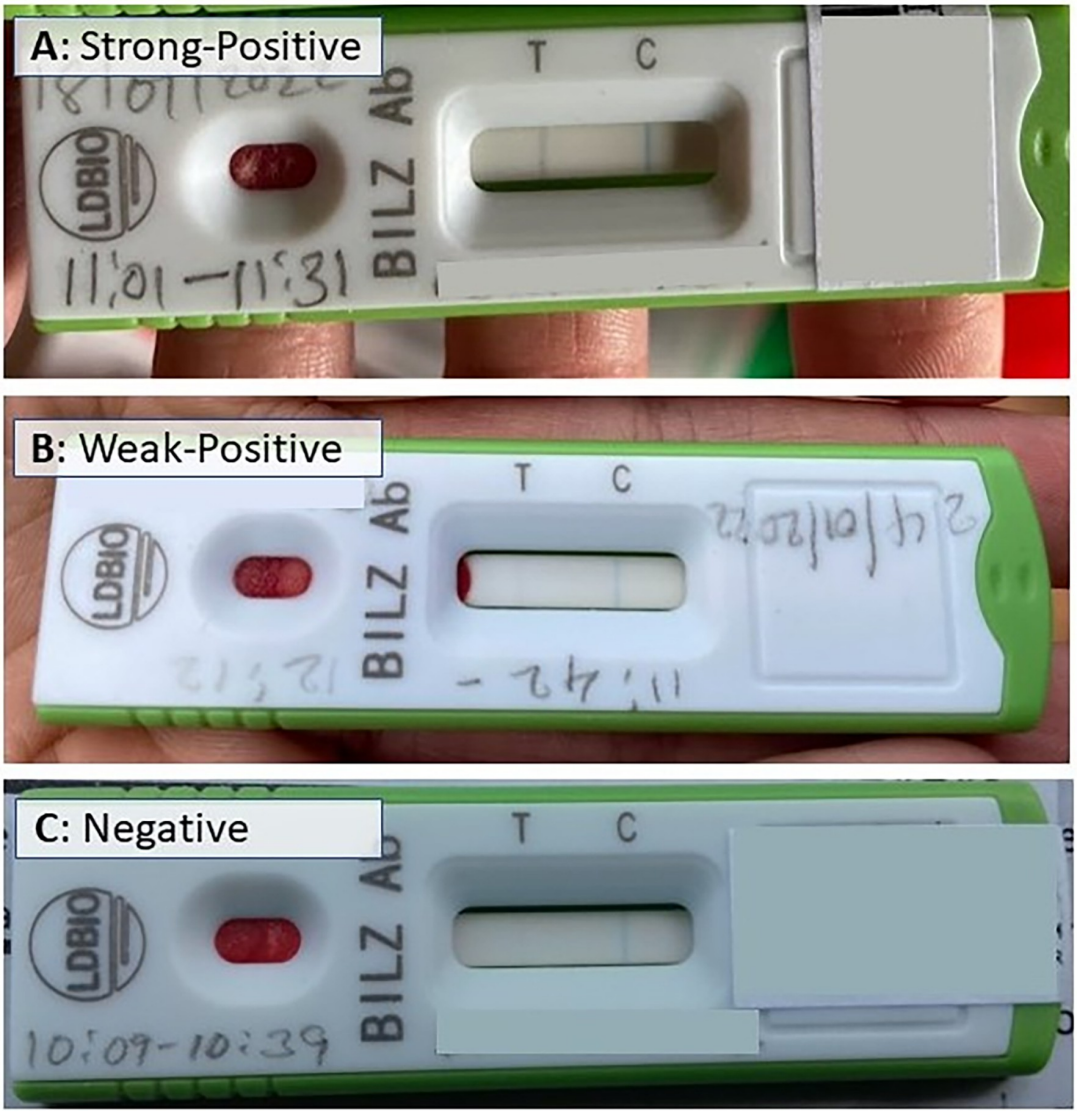
Example of *Schistosoma* immunochromatographic IgG-IgM rapid test classification: strong-positive test (A), weak-positive test (B), negative test (C).

The quantitative data for this study were obtained from a cross-sectional community-based survey that was nested in a four-arm, single blinded, parallel, cluster randomized control trial assessing the efficacy of three dual active-ingredients LLIN for the control of malaria [40]. Additional survey questions and a schistosomiasis testing component were added to data collection activities for the main malaria intervention trial to assess exposures that are independently and jointly associated with malaria and schistosomiasis. Data collection methods are described in detail elsewhere [40]; briefly, 45 households were randomly selected from each cluster using a census list generated at the trial baseline, and data were electronically captured using tablets/smartphones installed with ODK. Questionnaire data were collected in January 2022, three years after the trial’s distribution of LLINs, from approximately 1,300 head of households (HoHs), and one child per household between the ages of five and fourteen were randomly selected for malaria and schistosomiasis testing the following day. All selected children were tested for malaria parasitemia using malaria rapid diagnostic tests (mRDTs) (CareStart RDTs; HRP2, (pf), DiaSys, Wokingham, UK), and schistosomiasis exposure (does not distinguish between *S*. *mansoni* and *S*. *haematobium)* using immunochromatographic (ICT) IgG-IgM rapid tests (sRDTs) (LDBio Diagnostics Inc, Lyon, France). When mRDTs and sRDTs were positive, free artemether-lumefantrine and Praziquantel tablets were provided, respectively. The mRDTs were kept at ambient temperature in sealed bags with desiccant packs for up to six weeks before being transferred to 4°C where they were kept until September 2022. Pictures of all complete sRDTs were taken using tablets/smartphones and classified based on the intensity of the test band with more intense bands hypothesized to reflect higher IgM antibody titers (i.e., recent infection) relative to IgG antibody titers (i.e., past infection). A strong-positive test was characterized by a sRDT with a clear and distinct test band (recent infection) (Fig 2A), whereas a weak-positive test was characterized by a faint test band (past infection) (Fig 2B).

#### Environmental data

The environmental data for this study (temperature, precipitation, NDVI, population density and distance to Lake Victoria) were obtained from publicly accessible satellite imagery and are defined in the (S2 Text: Variable Definitions). Briefly, daytime land surface temperature (LST) and NDVI were obtained from the Moderate Resolution Imaging Spectroradiometer (MODIS) [43, 44], precipitation was obtained from WorldClim [45], population density was obtained from WorldPop [46], and distance to Lake Victoria was calculated using data from Natural Earth [47]. The finest spatial resolution available from each source were chosen including: 1km for LST, 250 m for NDVI, 1 km for population density and precipitation. These data were then linked to each household from the cross-sectional survey.

#### Performance of schistosomiasis rapid diagnostic test

The performance of the sRDTs were assessed by measuring the sensitivity and specificity of the test compared to a gold standard enzyme-linked immunosorbent assay (ELISA) kit. We retrieved and eluted blood spots from 188 (15%) mRDTs from the cross-sectional to detect anti-Schistosoma *mansoni* and *haematobium* IgG and IgM antibodies using Human Schistosoma *mansoni* Antibody IgM ELISA kits, Schistosoma *mansoni* Antibody IgG ELISA kits, and Schistosoma *haematobium* Antibody ELISA kits (MyBiosource Inc, San Diego, USA). The elution procedure followed the methods outlined by Williams et al., 2009 [48]. Briefly, the mRDTs were dismantled and the blood exposed areas were cut into small pieces and placed in a 1.5 mL microtubes immersed in: 1) 0.5mL Phosphate-buffered saline (Invitrogen), 2) 0.05% (v/v) Tween-20 (Sigma), and 3) 0.1% (w/v) Sodium Azide (Aldrich). The microtubes were agitated overnight, and the eluted solution was used as directed by the manuals outlined in the ELISA kits.

#### Community mapping

The qualitative data collection for this study was conducted in August 2022 through a community mapping activity combined with direct field observations. Two villages from the cross-sectional study (Isesa and Mwagimagi) were selected based on malaria, schistosomiasis, and co-infection prevalence (one with high prevalence for all and one with low prevalence for all), and proximity to Lake Victoria (one inland and one by the lake). At some point between the formation of the study area and the community mapping, Gukwa seceded from Mwagimagi to become a separate village–therefore, additional maps were created for Gukwa. Ten community members above the age of 18 from each village (at least 5 men and 5 women per village) were selected by village leaders to create a map of their village (one map for men, and one map for women) to identify the location of temporary and permanent bodies of water (which can serve as mosquito larval breeding sites and snail habitats) as well as their specific uses (i.e., bathing, laundry, fetching water). We then identified the features in the community maps, along with other bodies of water that were not identified in the map, to understand water access and use behaviors.

#### Statistical approach

Data analysis focused on three primary outcomes: 1.1) malaria prevalence, 1.2) schistosomiasis seroprevalence, and 1.3) malaria and schistosomiasis co-infection; and two secondary outcomes: 2.1) strong-positive schistosomiasis seroprevalence (based on picture classification) and 2.2) malaria and strong-positive schistosomiasis co-infection.

Candidate individual-level determinants, household-level social determinants and environmental determinants hypothesized to be associated with malaria, schistosomiasis, and co-infection are detailed below and further defined in a (S2 Text: Variable Definitions). To identify overlapping determinants across the two diseases and for comparability across the models, the same set of variables were included in all three models. Individual determinants, such as age and sex, are biological features that are often associated with disease. Social determinants include social and economic factors that may expose someone to malaria or schistosomiasis which include household socioeconomic status (SES), HoH education, HoH occupation, HoH knowledge of diseases, and HoH perception of the diseases. Environmental determinants include factors that create favorable conditions for malaria and schistosomiasis vectors and parasites and include climate, urbanization, and distance and exposure to breeding sites. A wealth score, as a proxy for household SES, was calculated based on household durable assets and dwelling characteristics using a Principal Component Analysis. The wealth score was ranked and categorized into quintiles where the first quintile represented the poorest group and the fifth quintile the least poor group [49, 50]. LLIN access is defined by the WHO as the percentage of population that could be protected by a LLIN, if each LLIN in a household could be used by two people [51]. The water, sanitation, and hygiene (WASH) indicators were dichotomized as either improved or unimproved sources as defined by the Joint Monitoring Programme for Water Supply, Sanitation and Hygiene [52]. A knowledge score was calculated based on spontaneously identifying at least one correct symptom and at least one mechanism of transmission for each disease [53, 54]. Perception of each disease was measured for the head of household by the question “in your opinion, how many people in your village have malaria/schistosomiasis?” with the following options: none, few, some, many, everyone, don’t know (was not read out as an option). The following categories were collapsed: few/some and many/everyone due to low response rates in some categories. Concern of each disease was measured for the head of household by the question “are you concerned about malaria/schistosomiasis personally?” with the following options: not at all concerned, slightly concerned, somewhat concerned, moderately concerned, extremely concerned, don’t know (was not read out as an option). The following categories were collapsed: somewhat to slightly concerned and moderately to extremely concerned due to low response rates in some categories. A households distance to Lake Victoria was categorized as near (<1km) and far (>5km) based on previously published definitions [25, 55].

#### Descriptive statistics

Descriptive statistics were used to summarize the characteristics of the study population. Frequencies and proportions for categorical variables and median, minimum, and maximum values for continuous variables were generated in SAS version 9.4 (SAS Institute, Cary, NC, USA). The differences in study population in the two selected villages for the community mapping (Mwagimagi and Gukwa are combined for this analysis) were compared using chi-square, Fisher’s exact, or a Kruskal-Wallis tests. P-values less than 0.05 were considered statistically significant.

#### Mixed-effect logistic regression models

Mixed effects logistic regression models accounting for a clustered sampling design were used to identify individual- and household-level social determinants, and environmental determinants that were associated with all five outcomes. The PROC GLIMMIX procedure in SAS version 9.4 (SAS Institute, Cary, NC, USA) was used to fit the generalized linear mixed model (GLMM) using the Laplace parameter approximation, a logit link, and random effects at the cluster level. A random effect term for the cluster in which the individual belonged was included to account for the heterogeneity in the 48 clusters–the random effect was assumed to follow a normal distribution. The models were also adjusted for the study intervention arm to account for potential biases or effects introduced by the type of LLIN assigned as part of the main trial protocol. Using SAS version 9.4 (SAS Institute, Cary, NC, USA), the univariable association between all candidate explanatory variables and each outcome was assessed, and variables with a P-value of less than 0.2 were entered into a multivariable model. For models that could not accommodate all candidate explanatory variables due to low numbers of events (co-infection secondary analysis), the “Dredge” function in the MuMIn package in RStudio version 4.1.3 (R Core Team, 2018) was used to determine the best combination of explanatory variables from those selected using a P-value of less than 0.2 [56]. The Dredge function is a model selection tool that ranks every candidate model (2n models, where n is the number of predictors in the full model) based on the Akaike Information Criterion (AIC). A limit of ten events per variable was placed for the model selection and the final model based on minimizing the AIC for the models using the “Dredge” function was fit.

Multicollinearity between explanatory variables in the mixed effects logistic regression models for all five outcomes was assessed–a variance inflation factor of greater than 2.5 was considered substantial collinearity [57]. Model fit was assessed in SAS version 9.4 (SAS Institute, Cary, NC, USA) by the area under a receiver operating characteristic curve (AUC) which is a measure of discrimination that evaluates the models’ ability to correctly classify cases and non-cases. AUC values range from 0.5 to 1, with higher values indicating better discrimination. AUC values between 0.5 and 0.7 indicated poor discrimination, values between 0.7 and 0.8 indicated acceptable discrimination, values between 0.8 and 0.9 indicated excellent discrimination and 0.9 or higher indicated outstanding discrimination [58]. Spatial autocorrelation was then assessed by computing the Global Moran’s I statistic, which measures the degree of spatial autocorrelation of the regression residuals, which was implemented in ArcMap version 10.8.1 (ESRI, Redlands, CA, USA) [59]. A P-value less than 0.05 was considered statistically significant, indicating the presence of significant spatial autocorrelation in the residuals.

#### Cluster analysis

Spatial clusters of high and low infection at the household-level for all five outcomes were assessed using Getis-Ord-Gi* in ArcMap version 10.8.1 (ESRI, Redlands, CA, USA) and further analyzed using SatScan software (v 9.4.1 Kulldorf and Information Management Services, Inc.) [60]. Getis-Ord-Gi* is a statistical technique used to identify hotspots (high value surrounded by high value) and coldspots (low value surrounded by low value) that are determined at the 99%, 95% and 90% confidence levels. For example, at a 5% level of significance, a Getis-Ord-Gi* z-score greater than 1.96 indicates statistically significant hotspots and values less than -1.96 indicate statistically significant coldspots. In this analysis, the data were analyzed at the household-level using the default settings (fixed distance band and a Euclidean distance between features) to identify hotspots and coldspots in the study area. SatScan is a software that detects clusters of high or low values in spatial data by using a scanning window of varying radii that moves across the study area. A relative risk for each cluster is then generated, where a risk ratio (RR) of greater than 1 represents a cluster where the probability of being an event within the scanning window is greater than the probability of being an event outside the scanning window (RR>1). A P-value less than 0.05 was considered statistically significant, indicating that the observed cluster is unlikely to have occurred by chance alone. Results from the Getis-Ord-Gi* and SatScan were overlaid in ArcMap and visually inspected to identify hotspots and coldspots of infection.

#### Qualitative approach

The community mapping activity combined with direct field observations were conducted to strengthen our understanding of the risk factors and exposures to malaria and schistosomiasis. We observed and engaged with individuals fetching water to understand their water use behaviors. Along with structured visual observations (initial plan determined by community maps), we took detailed notes on topics such as: 1) water use (i.e., drinking water, bathing water), 2) number of times a day the water source was frequented, and 3) the rationale for visiting a particular water source. Themes and subthemes from the community maps and field observations were used to support and explain the higher than hypothesized schistosomiasis seroprevalence in the study area–beyond what was captured by the questions in the cross-sectional survey.

#### Ethics statement

The protocol for this study was reviewed and approved by the institutional review boards, of the University of Ottawa (Canada) and Medical Research Coordinating Committee of the National Institute for Medical Research (Tanzania). Written informed consent forms were obtained from the HoH, adult guardian in the household, or participant prior to data collection activities. Study data did not include information that could be used to identify individual study participants. All participants were anonymized using study numbers as unique participant identifiers. Additional information regarding the ethical, cultural, and scientific considerations specific to inclusivity in global research is included in the (S3 Text: Inclusivity in Global Research).

## Results

### Characteristics of the study population

1122 children were included in this study. The median age of selected children was 9 years old, and there were an equal number of girls (n = 563, 50.2%) and boys (n = 559, 49.8%) (Table 1). Nearly two thirds of HoHs had a primary school education (n = 754, 67.2%) and most were farmers (n = 1055, 94.0%). LLIN ownership (households with at least 1 LLIN) was high (n = 1077, 96.0%), but adequate household-level LLIN access (1 LLIN for every 2 people in the household) was low (n = 314, 28.0%). The majority (81.1%) of households had unimproved sanitation facilities, with 17.6% (n = 198) of households without any toilet facility (bush toilets). The prevalence of malaria, seroprevalence of schistosomiasis, and co-infection, were 40.5%, 94.3%, and 38.1% respectively (Table 2). For the secondary analysis, the seroprevalence of strong-positive schistosomiasis and strong-positive co-infection in this study were 29.7% and 14.2%, respectively. Children in Mwagimagi had a higher malaria prevalence than Isesa (83.3% vs 30.6%, *P*<0.0001) and households in this village were further away from Lake Victoria compared to Isesa (100% of households in Mwagimagi >5km from Lake Victoria vs 44.9% households in Isesa >5 km from Lake Victoria, *P*<0.0001).

**Table 1:** Characteristics of the study participants and child infection/seropositivity status in the 48 clusters and the two selected villages for the qualitative activities in Misungwi district, Lake Zone, Tanzania, in January 2022.

		Total* (n = 1122)	Mwagimagi (n = 42)	Isesa (n = 49)	P-value^%^
**Child Infection**					
	Malaria	454 (40.5%)	35 (83.3%)	15 (30.6%)	<0.0001
	Schistosomiasis	1058 (94.3%)	38 (90.5%)	49 (100%)	0.0898
	Co-infection	428 (38.1%)	31 (73.8%)	15 (30.6%)	<0.0001
	Strong-positive Schistosomiasis^#^	328 (29.7%)	13 (31.7%)	14 (29.2%)	0.9772
	Strong-positive Co-infection^#^	156 (14.2%)	10 (24.4%)	7 (14.6%)	0.3667
**Individual Determinant (child)**					
Age of selected child (median)					
		9 (5, 14)	10 (5, 14)	10 (5, 14)	0.7122
Sex of selected child					
	Girl	563 (50.2%)	19 (45.2%)	30 (61.2%)	0.1888
	Boy	559 (49.8%)	23 (54.8%)	19 (38.8%)	
**Social Determinants (household)**					
Head of household education					
	None	318 (28.3%)	16 (38.1%)	11 (22.4%)	0.1894
	Primary	754 (67.2%)	26 (61.9%)	37 (75.5%)	
	Secondary or higher	50 (4.5%)	0	1 (2.1%)	
Head of household occupation					
	Farming	1055 (94.0%)	41 (97.6%)	48 (98.0%)	1
	Other	67 (6.0%)	1 (2.4%)	1 (2.0%)	
Socioeconomic status					
	Lowest	233 (20.8%)	12 (28.6%)	8 (16.3%)	0.6401
	Low	235 (20.9%)	10 (23.8%)	12 (24.5%)	
	Average	240 (21.4%)	7 (16.7%)	12 (24.5%)	
	High	202 (18.0%)	8 (19.0%)	9 (18.4%)	
	Highest	212 (18.9%)	5 (11.9%)	8 (16.3%)	
*Malaria-only*					
Knowledge of disease					
	Yes	882 (78.6%)	36 (85.7%)	41 (83.7%)	0.7879
	No	240 (21.4%)	6 (14.3%)	8 (16.3%)	
Perception of disease					
	None	18 (1.6%)	0	0	0.8507
	Few/some	346 (30.8%)	11 (26.2%)	14 (28.6%)	
	Many/everyone	462 (41.2%)	17 (40.5%)	17 (34.7%)	
	Don’t know	296 (26.4%)	14 (33.3)	18 (36.7%)	
Concern of disease personally					
	Not at all concerned	255 (22.7%)	3 (7.1%)	15 (30.6%)	0.0324
	Somewhat to slightly concerned	196 (17.5%)	6 (14.3%)	6 (12.2%)	
	Moderately to extremely concerned	642 (57.2%)	32 (76.2%)	28 (57.1%)	
	Don’t know	29 (2.6%)	1 (2.4%)	0	
LLIN Access					
	Yes	314 (28.0%)	8 (19.0%)	9 (18.4%)	1
	No	808 (72.0%)	34 (81.0%)	40 (81.6%)	
*Schistosomiasis-only*					
Knowledge of disease					
	Yes	419 (37.3%)	17 (40.5%)	17 (34.7%)	0.5698
	No	703 (52.7%)	25 (59.5%)	32 (65.3%)	
Perception of disease					
	None	99 (8.8%)	3 (7.1%)	3 (6.1%)	0.5221
	Few/some	450 (40.1%)	21 (50.0%)	19 (38.8%)	
	Many/everyone	141 (12.6%)	2 (4.8%)	6 (12.2%)	
	Don’t know	432 (38.5%)	16 (28.1%)	21 (42.9%)	
Concern of disease personally					
	Not at all concerned	375 (33.4%)	9 (21.4%)	4 (8.2%)	0.0022
	Somewhat to slightly concerned	197 (17.6%)	11 (26.2%)	4 (8.2%)	
	Moderately to extremely concerned	488 (43.5%)	17 (40.5%)	22 (44.9%)	
	Don’t know	62 (5.5%)	5 (11.9%)	0	
Drinking Source					
	Improved	245 (21.8%)	0	0	NA
	Unimproved	877 (78.2%)	42 (100%)	49 (100%)	
Sanitation facility					
	Improved	169 (15.1%)	0	6 (12.2%)	
	Pit latrine	755 (67.3%)	30 (71.4%)	38 (77.6%)	
	Bush toilet	198 (17.6%)	12 (28.6%)	5 (10.2%)	0.0094
Hygiene					
	Improved	751 (66.9%)	28 (66.7%)	32 (65.3%)	1
	Unimproved	371 (33.1%)	14 (33.3%)	17 (34.7%)	
**Environmental Determinants**					
Temperature (°C)					
		30.0 (22.7, 34.7)	30.0 (27.9, 31.5)	29.1 (23.8, 30.8)	<0.0001
Precipitation (mm)					
		98 (94, 103)	98 (96, 101)	99 (97, 101)	0.3073
NDVI					
	Sparse vegetation	972 (86.6%)	35 (83.3%)	39 (79.6%)	0.8519
	Dense vegetation	150 (13.4%)	7 (16.7%)	10 (20.4%)	
Population Density (per km^2^)					
	<100 per km^2^	128 (11.4%)	14 (33.3%)	2 (4.1%)	<0.0001
	100–200 per km^2^	642 (57.2%)	28 (66.7%)	15 (30.6%)	
	>200 per km^2^	352 (31.4%)	0	32 (65.3%)	
Distance to Lake Victoria (km)					
	Near (<1km)	87 (7.8%)	0	5 (10.2%)	<0.0001
	Middle	412 (36.7%)	0	22 (44.9%)	
	Far (>5km)	623 (55.5%)	41 (100%)	22 (44.9%)	

Data are displayed as n (%), median (min, max)

* Includes Mwagimagi and Isesa

%P-values (significant differences between two selected villages for community mapping, by using the chi squared test or fisher exact test for comparison of proportions, and Kruskal-Wallis test for medians.

# Secondary analysis based on picture classification where a strong-positive test was characterized by a sRDT with a clear and distinct test band (recent infection), and a weak-positive test was characterized by a faint test band (past infection).

**Abbreviations:** LLIN: Long Lasting Insecticidal Nets; LST: Land Surface Temperature; NDVI: Normalized Difference Vegetation Index

**Table 2:** Results of primary multivariable mixed effects logistic regression analysis of factors associated with malaria, schistosomiasis, and co-infection (n = 1122).

		Malaria			Schistosomiasis			Co-infection		
		aOR*	(95% CI)	P-value	aOR*	(95% CI)	P-value	aOR*	(95% CI)	P-value
Significant Determinants										
Age of Selected Child										
		1.19	(1.13–1.25)	<0.0001	1.26	(1.12–1.41)	<0.0001	1.20	(1.14–1.26)	<0.0001
Sex of Selected Child										
	Boy	1.44	(1.10–1.88)	0.0083	1.64	(0.90–3.01)	0.1080	1.54	(1.18–2.02)	0.0016
	Girl	REF	-	-	REF	-	-	REF	-	-
LLIN Access										
	No	1.67	(1.22–2.30)	0.0013	1.94	(1.03–3.62)	0.0389	1.67	(1.21–2.29)	0.0015
	Yes	REF	-	-	REF	-	-	REF	-	-
Knowledge of disease (schistosomiasis)										
	Yes	0.69	(0.51–0.94)	0.0167	-	-	-	0.71	(0.53–0.97)	0.0308
	No	REF	-	-	-	-	-	REF	-	-
Concern of disease personally (schistosomiasis)										
	Not at all concerned	0.67	(0.48–0.92)	0.0150	-	-	-	0.96	(0.50–1.86)	0.0324
	Somewhat to slightly concerned	0.68	(0.46–1.00)	0.0508	-	-	-	1.75	(1.09–2.82)	0.0057
	Don’t know	0.58	(0.31–1.12)	0.1035	-	-	-	1.80	(1.08–2.99)	0.1099
	Moderately to extremely concerned	REF	-	-	-	-	-	REF	-	-
Drinking water										
	Unimproved	2.02	(1.38–2.97)	0.0003	-	-	-	1.96	(1.35–2.86)	0.0005
	Improved	REF	-	-	-	-	-	REF	-	-
Population density										
	<100 per km^2^	2.59	(1.49–4.51)	0.0008	-	-	-	2.40	(1.41–4.08)	0.0012
	100–200 per km^2^	2.04	(1.41–2.95)	0.0002	-	-	-	2.10	(1.47–3.00)	<0.0001
	>200 per km^2^	REF	-	-	-	-	-	REF	-	-
Distance to Lake Victoria (km)										
	Middle	1.10	(0.61–1.98)	0.7346	-	-	-	1.04	(0.56–1.82)	0.9622
	Far (>5km)	1.84	(1.01–3.32)	0.0444	-	-	-	1.68	(0.95–2.96)	0.0718
	Near (<1km)	REF	-	-	-	-	-	REF	-	-
Non-Significant Determinants										
Socioeconomic status										
	Lowest	1.30	(0.81–2.07)	0.2794	-	-	-	1.21	(0.75–1.94)	0.4316
	Low	1.52	(0.96–2.41)	0.0738	-	-	-	1.51	(0.95–2.38)	0.0818
	Average	1.00	(0.64–1.48)	0.9857	-	-	-	0.96	(0.61–1.52)	0.8738
	High	0.95	(0.59–1.54)	0.8570	-	-	-	0.92	(0.57–1.49)	0.7342
	Highest	REF	-	-	-	-	-	REF	-	-
Perception of disease (malaria)										
	None	0.99	(0.34–2.88)	0.9946	0.32	(0.04–2.45)	0.2749	1.11	(0.38–3.21)	0.8411
	Few/some	1.01	(0.91–1.95)	0.9533	1.17	(0.56–2.40)	0.6782	1.05	(0.74–1.47)	0.7859
	Don’t know	1.33	(0.91–1.95)	0.1404	2.13	(0.85–5.35)	0.1061	1.32	(0.91–1.94)	0.1399
	Many/everyone	REF	-	-	REF	-	-	REF	-	-
Concern of disease personally (malaria)										
	Not at all concerned	-	-	-	1.68	(0.72–3.90)	0.2260	-	-	-
	Somewhat to slightly concerned	-	-	-	1.67	(0.64–4.40)	0.2955	-	-	-
	Don’t know	-	-	-	0.16	(0.04–0.68)	0.0136	-	-	-
	Moderately to extremely concerned	-	-	-	REF	-	-	-	-	-
Perception of disease (schistosomiasis)										
	None	0.81	(0.43–1.55)	0.5234	1.22	(0.38–3.86)	0.7352	0.70	(0.51–0.97)	0.9259
	Few/some	1.46	(0.92–2.32)	0.1101	1.59	(0.68–3.73)	0.2805	0.57	(0.38–0.85)	0.0213
	Don’t know	1.40	(0.85–2.30)	0.1833	1.83	(0.73–4.57)	0.1923	0.59	(0.31–1.12)	0.0230
	Many/everyone	REF	-	-	REF	-	-	REF	-	-
Sanitation facility										
	Pit latrine	1.14	(0.73–1.80)	0.5777	-	-	-	1.15	(0.72–1.83)	0.5535
	Bush Toilet	1.18	(0.68–2.04)	0.5557	-	-	-	1.34	(0.65–1.98)	0.6549
	Improved	REF	-	-	-	-	-	REF	-	-
Hygiene										
	Unimproved	1.06	(0.79–1.42)	0.6980	-	-	-	1.10	(0.82–1.48)	0.5211
	Improved	REF	-	-	-	-	-	REF	-	-

* Random effects for cluster and adjusted for intervention arm

- Variable was not included in the final model based on model selection criteria

**Abbreviations:** aOR: Adjusted Odds Ratio; LLIN: Long Lasting Insecticidal Nets; LST: Land Surface Temperature; NDVI: Normalized Difference Vegetation Index

### Determinants of malaria, schistosomiasis, and co-infection

The univariable association between all factors are included as a (S4 Text: Univariable Associations) and the results from the multivariable mixed effects logistic regression models are outlined in Table 2. Individual determinants, including age and sex of the selected child, emerged as the primary determinants driving the association with infection status. Older age was associated with higher infection prevalence for malaria (aOR = 1.19; *P*<0.0001), schistosomiasis (aOR = 1.26; *P*<0.0001), and co-infection (aOR = 1.20; *P*<0.0001); while boys had higher odds of infection compared to girls for malaria (aOR = 1.44; *P* = 0.0083), and co-infection (aOR = 1.54; *P* = 0.0016). A limited number of household-level social determinants were found to be associated with infection status. Not having adequate LLIN access was positively associated with malaria (aOR = 1.67; *P* = 0.0013), schistosomiasis (aOR = 1.94; *P* = 0.0389), and co-infection (aOR = 1.67; *P* = 0.0015) and having unimproved water sources was positively associated with malaria (aOR = 2.02; *P* = 0.0003) and co-infection (aOR = 1.96; *P* = 0.0005). Two environmental determinants, namely population density and distance to Lake Victoria, were key factors positively associated with malaria and co-infection, but not with schistosomiasis. Households in less densely populated areas (<100 compared to >200 people per km^2^) had higher odds of malaria infection (aOR = 2.59; *P* = 0.0008) and co-infection (aOR = 2.40; *P* = 0.0012), and households further from the lake (≥ 5km compared to <1km from the lake) had higher odds of malaria infection (aOR = 1.84; *P* = 0.0444). The final multivariable models for malaria and co-infection had acceptable discrimination (malaria: AUC = 0.76, co-infection: AUC = 0.75), while the model for schistosomiasis had outstanding discrimination (AUC = 0.91). There was no evidence of spatial autocorrelation for any primary model (malaria: *P* = 0.4120, schistosomiasis: *P* = 0.9796, co-infection: *P* = 0.3947) based on Global Moran’s I test.

Results from the primary analysis were comparable to results obtained from the secondary analysis that investigated factors associated with strong-positive schistosomiasis seropositivity and strong-positive co-infection, in terms of identified determinants and associated strength of association. Individual-level social determinants continued to be the primary determinants driving the association with strong-positive schistosomiasis seropositivity and strong-positive co-infection. Aligned with the primary analysis, older age was associated with higher prevalence for strong-positive schistosomiasis seropositivity (aOR = 1.20; *P*<0.0001), and strong-positive co-infection (aOR = 1.28; *P*<0.0001); while boys had higher odds compared to girls for strong-positive schistosomiasis seropositivity (aOR = 2.56; *P*<0.0001), and strong-positive co-infection (aOR = 2.46; *P*<0.0001) (Table 3). Differences in the two analyses were present for the association between LLIN access; unlike the primary analysis that indicated an association with co-infection prevalence and not schistosomiasis seroprevalence, the secondary analysis demonstrated a positive association between inadequate LLIN access and strong-positive schistosomiasis seropositivity (aOR 1.43; *P* = 0.0342) as well as strong-positive co-infection (aOR = 1.93; *P* = 0.0043). The final multivariable models for the secondary analyses had acceptable discrimination (strong-positive schistosomiasis: AUC = 0.77, strong-positive co-infection: AUC = 0.78). There was no evidence of spatial autocorrelation for any secondary model (strong-positive schistosomiasis: *P* = 0.9626, strong-positive co-infection: *P* = 0.9662) based on Global Moran’s I test.

**Table 3:** Results of secondary multivariable mixed effects logistic regression analysis of factors associated with malaria infection, strong-positive schistosomiasis seropositivity, and malaria and strong-positive schistosomiasis co-infection (n = 1122).

		Malaria			Schistosomiasis			Co-infection		
		aOR*	(95% CI)	P-value	aOR*	(95% CI)	P-value	aOR*	(95% CI)	P-value
Significant Determinants										
Age of Selected Child										
		1.19	(1.13–1.25)	<0.0001	1.20	(1.13–1.27)	<0.0001	1.28	(1.19–1.38)	<0.0001
Sex of Selected Child										
	Boy	1.44	(1.10–1.88)	0.0083	2.56	(1.91–3.43)	<0.0001	2.46	(1.68–3.61)	<0.0001
	Girl	REF	-	-	REF	-	-	REF	-	-
Knowledge of disease (malaria)										
	Yes	-	-	-	0.61	(0.43–0.87)	0.0073	-	-	-
	No	-	-	-	REF	-	-	-	-	-
LLIN Access										
	No	1.67	(1.22–2.30)	0.0013	1.43	(1.03–2.01)	0.0342	1.93	(1.23–3.02)	0.0043
	Yes	REF	-	-	REF	-	-	REF	-	-
Knowledge of disease (schistosomiasis)										
	Yes	0.69	(0.51–0.94)	0.0167	-	-	-	-	-	-
	No	REF	-	-	-	-	-	-	-	-
Drinking water										
	Unimproved	2.02	(1.38–2.97)	0.0003	-	-	-	-	-	-
	Improved	REF	-	-	-	-	-	-	-	-
Population density										
	<100 per km^2^	2.59	(1.49–4.51)	0.0008	0.94	(0.49–1.82)	0.8598	1.98	(0.97–4.05)	0.0600
	100–200 per km^2^	2.04	(1.41–2.95)	0.0002	1.53	(1.01–2.31)	0.0427	2.83	(1.74–4.59)	<0.0001
	>200 per km^2^	REF	-	-	REF	-	-	REF	-	-
Distance to Lake Victoria (km)										
	Middle	1.10	(0.61–1.98)	0.7346	0.65	(0.32–1.33)	0.2400	-	-	-
	Far (>5km)	1.84	(1.01–3.32)	0.0444	0.46	(0.20–1.06)	0.0707	-	-	-
	Near (<1km)	REF	-	-	REF	-	-	-	-	-
Non-Significant Determinants										
Socioeconomic status										
	Lowest	1.30	(0.81–2.07)	0.2794	-	-	-	-	-	-
	Low	1.52	(0.96–2.41)	0.0738	-	-	-	-	-	-
	Average	1.00	(0.64–1.48)	0.9857	-	-	-	-	-	-
	High	0.95	(0.59–1.54)	0.8570	-	-	-	-	-	-
	Highest	REF	-	-	-	-	-	-	-	-
Perception of disease (malaria)										
	None	0.99	(0.34–2.88)	0.9946	0.98	(0.32–2.99)	0.9747	-	-	-
	Few/some	1.01	(0.91–1.95)	0.9533	1.66	(1.18–2.35)	0.0036	-	-	-
	Don’t know	1.33	(0.91–1.95)	0.1404	1.37	(0.94–2.00)	0.0976	-	-	-
	Many/everyone	REF	-	-	REF	-	-	-	-	-
Concern of disease personally (malaria)										
	Not at all concerned	-	-	-	-	-	-	-	-	-
	Somewhat to slightly concerned	-	-	-	-	-	-	-	-	-
	Moderately to extremely concerned	-	-	-	-	-	-	-	-	-
	Don’t know	-	-	-	-	-	-	-	-	-
Perception of disease (schistosomiasis)										
	None	0.81	(0.43–1.55)	0.5234	-	-	-	1.31	(0.48–3.59)	0.5951
	Few/some	1.46	(0.92–2.32)	0.1101	-	-	-	2.78	(1.31–5.90)	0.0079
	Don’t know	1.40	(0.85–2.30)	0.1833	-	-	-	2.25	(1.05–4.81)	0.0351
	Many/everyone	REF	-	-	-	-	-	REF	-	-
Concern of disease personally (schistosomiasis)										
	Not at all concerned	0.67	(0.48–0.92)	0.0150	-	-	-	-	-	-
	Somewhat to slightly concerned	0.68	(0.46–1.00)	0.0508	-	-	-	-	-	-
	Don’t know	0.58	(0.31–1.12)	0.1035	-	-	-	-	-	-
	Moderately to extremely concerned	REF	-	-	-	-	-	-	-	-
Sanitation facility										
	Pit latrine	1.14	(0.73–1.80)	0.5777	-	-	-	-	-	-
	Bush Toilet	1.18	(0.68–2.04)	0.5557	-	-	-	-	-	-
	Improved	REF	-	-	-	-	-	-	-	-
Hygiene										
	Unimproved	1.06	(0.79–1.42)	0.6980	-	-	-	-	-	-
	Improved	REF	-	-	-	-	-	-	-	-
Environmental determinants										
Temperature (°C)										
		-	-	-	0.94	(0.84–1.07)	0.3940	-	-	-

* Random effects for cluster and adjusted for intervention arm

- Variable was not included in the final model based on model selection criteria

**Abbreviations:** aOR: Adjusted Odds Ratio; LLIN: Long Lasting Insecticidal Nets; LST: Land Surface Temperature; NDVI: Normalized Difference Vegetation Index

### Spatial patterns and hotspots of malaria, schistosomiasis, and co-infection

**Fig 3:**
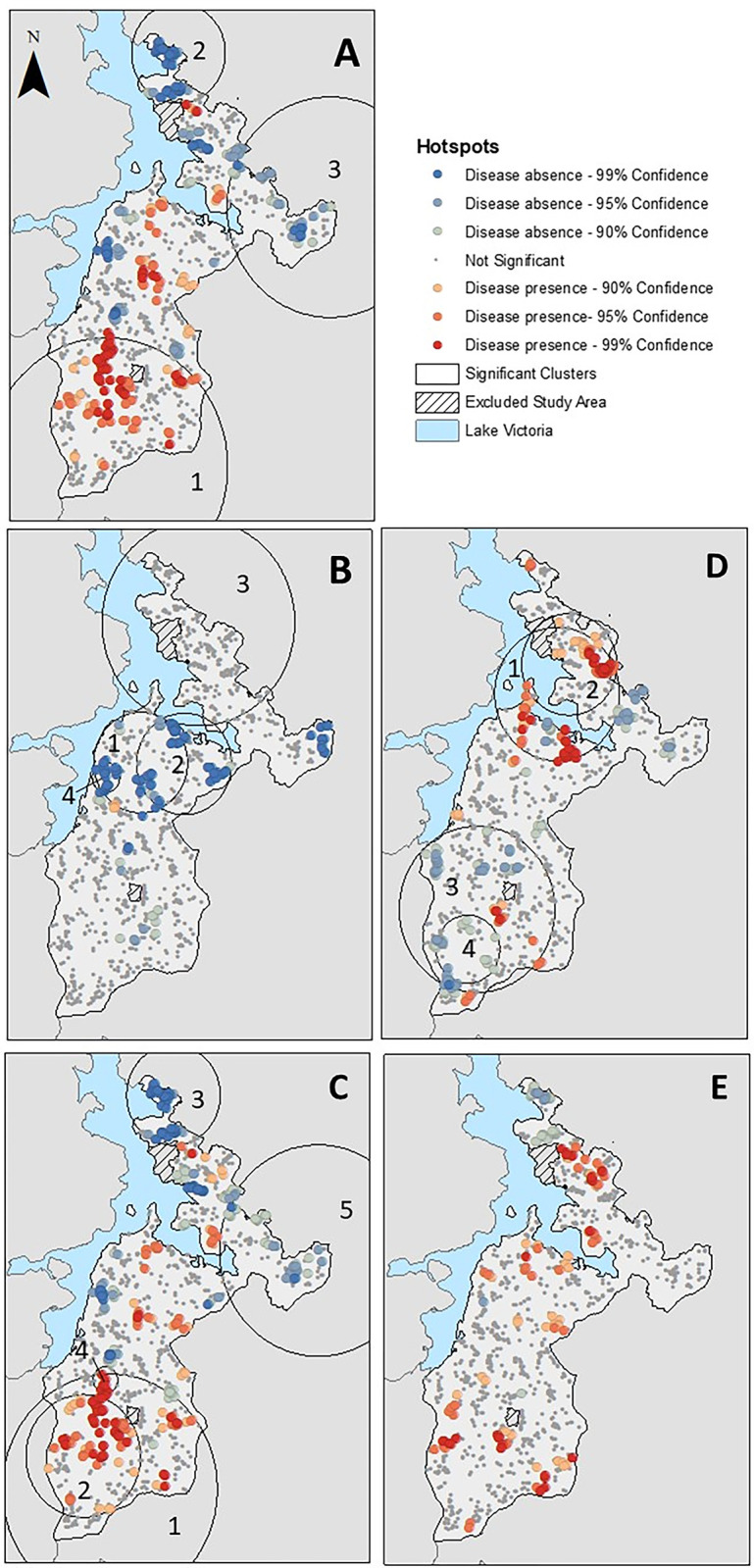
Spatial clusters of malaria prevalence (A), schistosomiasis seroprevalence (B), malaria and schistosomiasis co-infection (C), strong-positive schistosomiasis seroprevalence (D) and malaria and strong-positive schistosomiasis co-infection (E). Map content was produced with Esri ArcGIS software using study data and data provided by GADM available online: https://gadm.org/download_country.html.

There were geographic variations in infection prevalence and seroprevalence in the study area. Significant hotspots (defined visually and numerically based on overlayed results from Getis-Ord-Gi* and SatScan) of children with malaria were identified in the southern region of the study area (Cluster 1: RR = 1.68; *P*<0.001), while two significant coldspots were identified in the northern region of the study area (Cluster 2: RR = 0.07; *P*<0.001—Cluster 3: RR = 0.44; *P* = 0.022) (Fig 3A). Although there were statistically significant clusters of schistosomiasis infection in the study area, the spatial variation in seroprevalence was not clear with evidence of coldspots of infection along the inlet of Lake Victoria (Cluster 1: RR = 0.84; *P*<0.001—Cluster 2: RR = 0.83; *P*<0.001) (Fig 3B). The spatial patterning of co-infection followed that of malaria with significant hotspots and coldspots in the southern and northern region of the study area, respectively (Fig 3C).

The primary (malaria, schistosomiasis, and co-infection) and secondary (strong-positive schistosomiasis, and strong-positive co-infection) analysis both identified comparable factors associated with child infection status, however the spatial patterns in disease prevalence and seroprevalence exhibited notable differences between the primary and secondary analyses. There were two significant hotspots of strong-positive schistosomiasis seropositivity (Cluster 1: RR = 1.47; *P* = 0.001—Cluster 2: RR = 1.53; *P* = 0.012) (Fig 3D) in the inlet of Lake Victoria, where coldspots were previously identified in the primary analysis. There were also no significant clusters of strong-positive co-infection in the secondary analysis, compared to five that were identified in the primary analysis (Fig 3E).

### Performance of schistosomiasis rapid diagnostic test

The results from the Human *S*. *mansoni* Antibody IgM ELISA test, IgG ELISA test, and *S*. *haematobium* Antibody ELISA test using eluate from mRDTs collected during the cross-sectional survey, were all negative–likely reflecting the methods used to elute Schistosoma antibodies from the mRDTs.

### Community mapping and field observations

Six maps were created in the three selected villages (Isesa, Mwagimagi and Gukwa) (S5 Text: Six Community Maps). Community maps identified many unimproved water sources in both villages that were used by humans, cattle, or both. Overall, there were four themes that may provide an explanation for the higher than hypothesized schistosomiasis seroprevalence observed during the cross-sectional survey (Table 4).

**Table 4:** Emerging themes from community maps to understand and explain high schistosomiasis seroprevalence observed in children in Misungwi, Tanzania, during January 2022 cross-sectional survey.

Theme	Supporting Evidence
1. Ponds and wells	• Ponds and wells were sourced from the same body of water, separated by an arbitrary border.
	• Community members fetch drinking water from wells and use ponds (shared by cattle) for other domestic uses including bathing and washing.
2. Hand pumps	• Working hand pumps sourced from wells or boreholes were available in all three villages, but were not in use because of unpleasant taste and cost.
3. Seasonality	• Landscapes and sources of water can look different during rainy and dry season in all three villages.
4. Health-centres	• Malaria diagnosis and treatment using RDTs and antimalarials were available in all three villages. There were no control strategies for schistosomiasis outside of annual school-based MDA.

#### Ponds and wells

**Fig 4:**
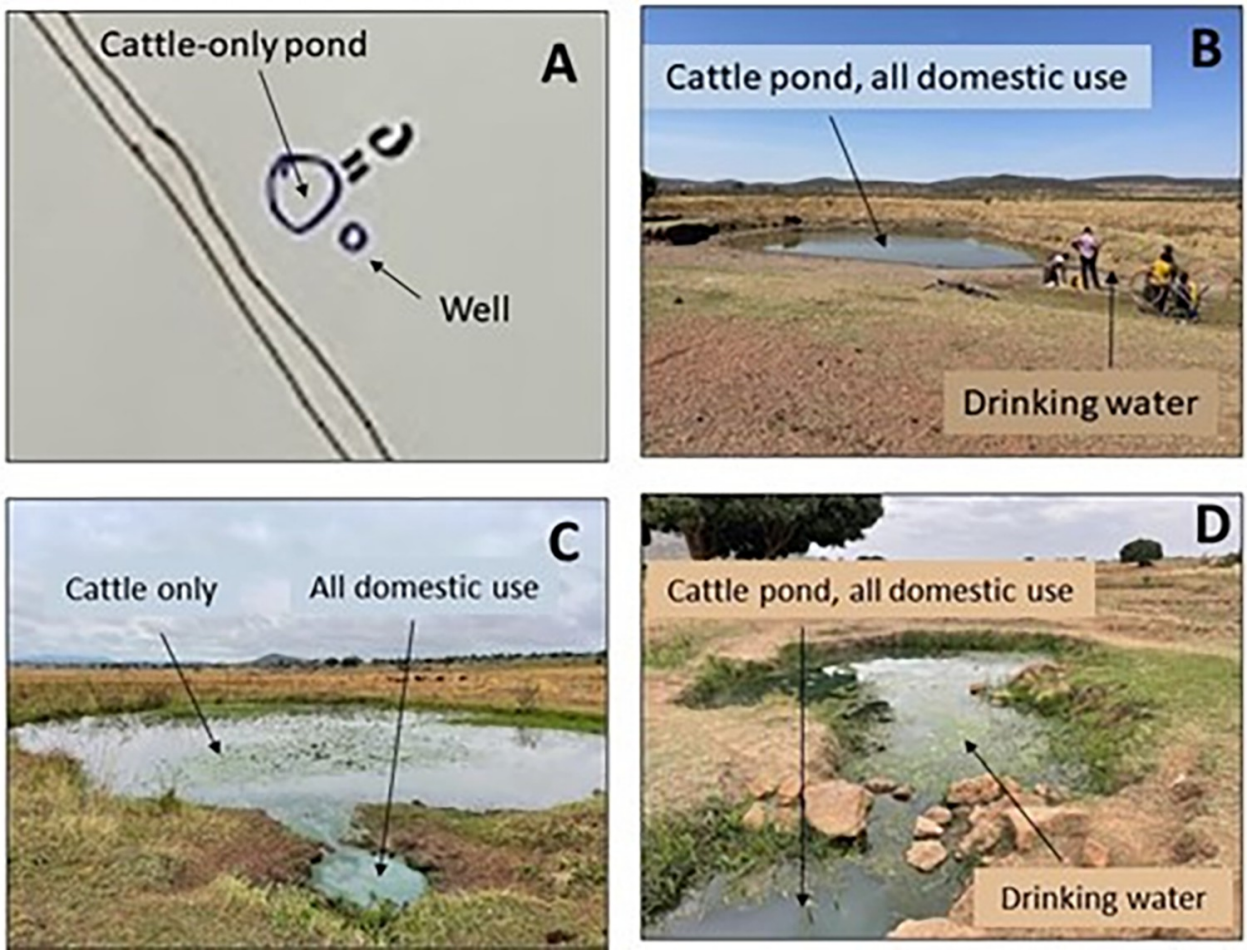
Cattle pond and well identified in community map (A), pond and well in Mwagimagi identified in the village (B), pond and well in Gukwa identified in the village (C), pond and well in Isesa identified in the village (D).

Community maps identified many water sources, often noting ponds next to wells (Fig 4A) Some maps made the distinction between cattle-only and human-only water sources, while others did not. In observing and interacting with the community map features, we found that ponds and wells were sourced from the same body of water separated by size and an arbitrary border, with the ponds being the larger body of water of the two (Fig 4B–4D). Wells were exclusively used for drinking water, while ponds were typically used by cattle and for all domestic uses (i.e., bathing, washing, playing). Children and women were often noted fetching water from an open water source (primarily unprotected wells) with jerry cans, up to four-times a day, while boys were found playing in the ponds.

#### Hand pumps

Only women in Isesa identified a hand pump as a water source in their community map. We found multiple working hand pumps sourced from either wells or boreholes in all three villages, however they were not in use because of unpleasant taste and cost. The hand pumps in both villages cost roughly 50 TSH (converted to $0.02 USD) for 20 litres of water. The quality and safety of the water sourced from the hand pumps were not assessed by the research team.

**Fig 5:**
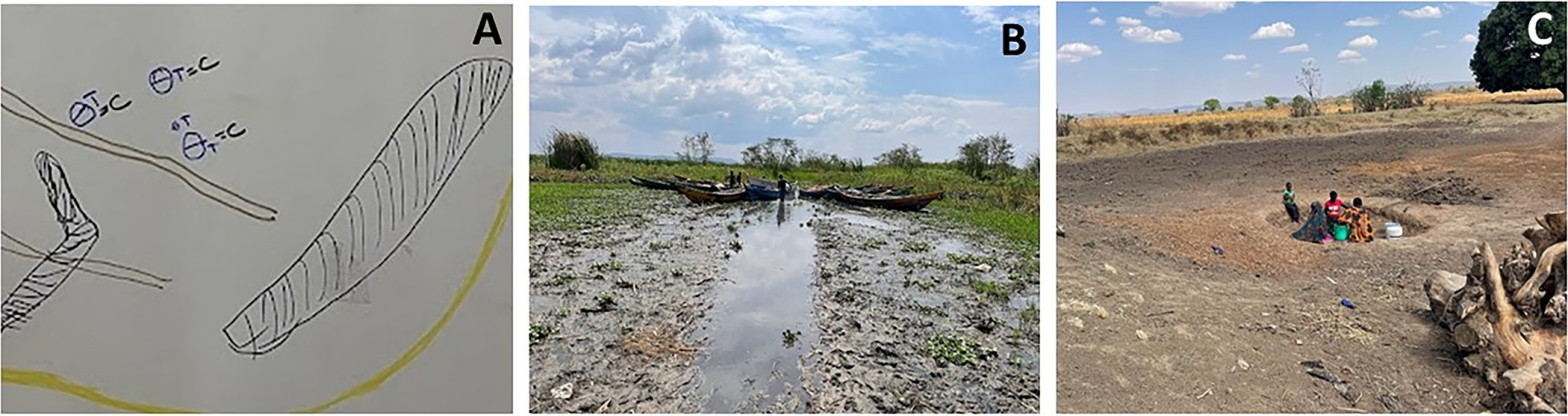
Temporary and permanent water sources identified in male community map in Mwagimagi (A), area along Lake Victoria during dry season in Isesa (B), dry pond during dry season in Mwagimagi (C).

**Seasonality.** All six community maps identified temporary and permanent water sources in their community maps (Fig 5A). The community mapping and field observations were conducted during the dry season before the Vuli (short rains from October to January). Community members from Isesa noted that the border of Lake Victoria extends up to 50-meters inland during the rainy season (Fig 5B). We also found that some riverbeds and ponds were dry and were further informed that water levels can reach one meter deep during the rainy season (Fig 5C). Some residents also noted that rainfall provides additional sources of water for their household during the rainy seasons.

#### Health-centres

Community maps drawn by women and men in Isesa, Mwagimagi, and drawn by women in Gukwa identified a health centre in their village. There were two health centres in Isesa, one in Mwagimagi, and none in Gukwa. Malaria RDTs and antimalarials were present at the health centres, but there were no schistosomiasis testing capacities nor Praziquantel available in the three villages, including pharmacies. Pharmacies were often noted to also have inconsistent working hours.

## Discussion

This mixed-method study assessed risk factors for malaria and schistosomiasis among children in Misungwi, Lake Victoria zone, Tanzania, an area of co-endemicity. The prevalence of malaria infection, seroprevalence of schistosomiasis, and co-infection in this study were 40.4%, 94.3%, and 38.1%, respectively. Individual-level factors emerged as the primary determinants driving the association with infection status, with age and sex (boys vs girls) being statistically and positively associated with malaria, schistosomiasis, and co-infection outcomes. Despite efforts to examine household-level social determinants and environmental determinants, a limited number of factors were found to be associated with infection prevalence and seroprevalence which could be attributed to the high levels of exposures to mosquito larval breeding sites and snail habitats in the study area. The widespread exposure to malaria and schistosomiasis diminishes the association and distinction between social and environmental factors such as socioeconomic status or temperature with infection prevalence and seroprevalence since everyone is already exposed and vulnerable to infection. Community maps identified unimproved water sources in all three villages that were used by humans, cattle, or both. We found that children primarily fetched water, and that unprotected wells were dedicated for drinking water whereas ponds were dedicated for other domestic uses and cattle.

The results from this study suggest that malaria and schistosomiasis share a common population at risk, and that older boys are particularly vulnerable to both diseases which is consistent with other studies [25, 29]. The relationship between age and schistosomiasis infection likely reflects prolonged exposures to contaminated water (i.e., fetching watching with jerry cans, bathing, laundry) in older compared to younger age groups, while the sex disparity likely reflects gender norms and water contact activities [61, 62]. We found that boys were exposed to schistosomiasis by playing in potentially contaminated water and exposed to malaria by herding cattle and ensuring they grazed and had access to water.

Neither knowledge nor perception questions regarding malaria and schistosomiasis were found to have a significant association with infection prevalence and seroprevalence, likely reflecting exposures to both diseases. For instance, although housing structure was included in the wealth score as a proxy for household SES, we found that majority of households had unimproved housing (eaves: 31.6%; grass/leaf roofs: 24.6%) that could increase the risk of a child’s exposure to malaria by allowing mosquitoes to enter their home [12, 63]. It is also important to note that most of the households were exposed to potentially contaminated water that can serve as mosquito larval breeding sites and snail habitats. This was evidenced by the community walkthrough and the high percentage of households with unimproved sources of water (78.2%) and sanitation facilities (84.9%). Considering these results, 2 of the 17 Sustainable Development Goals specifically state that everyone (100%) should have access to safe and affordable water and sanitation coverage by 2030 (SDG 6), and that we should end the epidemics of malaria and schistosomiasis (among others diseases) (SDG 3) [64]. It is evident that we are facing significant challenges in meeting both the Sustainable Development Goal on clean water and sanitation and on good health and well-being, which not only have serious implications for malaria and schistosomiasis, but to other water-borne diseases and neglected tropical diseases as well.

Another key factor to consider regarding risk factors for malaria and schistosomiasis and the Sustainable Development Goals, are that cattle are also present in and around human water sources in this study area. Cattle are not considered to be a significant source of transmission of schistosomiasis in SSA (intestinal and urogenital), but there is evidence of a hybrid species of *S*. *haematoboum* and *S*. *bovis* (the livestock *Schistosome*) in west Africa [65]. It is crucial to consider a One Health approach such as implementing environmental control and/or access to clean water and sanitation strategies, in high transmission areas such as Misungwi, to prevent even further public health problems.

The results of our analysis support the consideration of environmental control measures in high transmission areas like Misungwi, such as access to safe water and sanitation (for malaria and schistosomiasis), larval source management (for malaria), and snail control (for schistosomiasis), given the potential added benefits. Larval source management including mosquito larval habitat modifications, habitat manipulations, biological control, and larviciding, targets the immature stage of the *Anopheles* mosquitoes, whereas LLINs target the adult stage–thereby making these effective and complementary approaches [66]. Although larval source management is only recommended where larval breeding sites are few, findable and fixed, it may be beneficial to investigate this integrated approach with LLIN distribution in high transmission areas like Misungwi to reduce malaria transmission [66]. Similarly for snail control for schistosomiasis, snail populations can be greatly reduced with molluscicide application, but they are rarely eliminated [17]. MDA are crucial in controlling schistosomiasis, but it is evident that there is a need to more holistically integrate environmental control strategies to reduce exposures to and transmission of schistosomiasis.

The WHO recommends that Praziquantel be available in health facilities for the treatment of schistosomiasis [19, 67], and a study conducted in North-western Tanzania in 2021 found that 91.3% of the public health facilities had Praziquantel tablets available to patients [68]. However, we found that Praziquantel was not available outside of the school-based MDA (in dispensaries or health centres) in the three selected villages. In Tanzania, school-aged children are targeted for MDA with the goal of reaching at least 75% of the population at risk for schistosomiasis [22]. Despite a current endemicity of ≥50%, there have been more than five effective rounds of MDA (>75% population covered) in Tanzania, with a national coverage of 94%, 47%, and 65%, in 2021, 2020, and 2019, respectively [22, 69]. Having completed five effective rounds of MDA (based on population coverage), a recent modelling study by Li et al., revealed that while there is a reduction in prevalence during the first two rounds of MDA, the prevalence can level off during subsequent cycles [70]. In this study, we found that almost all children had been exposed to schistosomiasis at least once in their life (seroprevalence of 94.3%). Praziquantel does not prevent reinfection, but it prevents disease progression and reduces the risk of transmission to others by reducing the number of mature *Schistosome* worms in the host [71, 72]. Studies have found that there are stark decreases in the number of worms four-weeks after the administration of Praziquantel, and that the number of worms return to initial levels after six-months, in high transmission areas [73, 74]. Although repeated MDA poses a greater threat to Praziquantel resistance, biannual MDA combined with environmental control may be more effective than annual MDA at reducing the prevalence of schistosomiasis in persistent and high-transmission areas such as Misungwi [22, 75].

This study was nested in an ongoing four-arm, single blinded, parallel, cluster randomized control trial assessing the efficacy of three dual active-ingredients LLIN for the control of malaria. Although the LLIN metrics may not be generalizable to the entire population of Tanzania, it highlights important limitations in the availability and behaviors surrounding the main malaria vector control tool. Usual malaria vector control prevention strategies are based on a three year LLIN campaign targeting one LLIN for every two people [2]. In Tanzania, the national average for household-level LLIN ownership and access are 78% and 63%, respectively [76]. In our study, we found that while LLIN ownership within the household was high (96.0%), access to LLIN was limited (28.0%), for unspecified reasons. These results suggest that LLIN, as per the WHO’s definition (one LLIN for two people [51]), may not align with actual practices, where individuals might find themselves sharing LLIN with three or more people. While the models did not identify any spatial autocorrelation, there were hotspots of malaria prevalence in the study area, namely in the southern area of the study area, which may indicate the need for local strategies and targeted interventions. These hotspots could be attributed to unmeasured determinants or the use of publicly available environmental data which are not available at a fine spatial resolution (the spatial resolution of the data utilized in this study ranged between 250 meters and 1 kilometer).

Insecticide resistance in *Anopheles* mosquitoes, coupled with their behavioral resistance, also poses a threat to current efforts aimed at controlling malaria. Current control measures for malaria (LLIN) are most effective against mosquito species that are both anthropophilic (prefer to take their blood meals from human compared to other animals) and endophagic (feed indoors). Studies have however found that after LLIN distribution or community wide LLIN use, the mosquito vectors have the ability to alter their behavior by either feeding earlier [77] or shifting from endophilic to exophagic tendencies [78, 79] to feed on human hosts while they are not using LLINs. There is also evidence of shifts in malaria vector species to those that are exophagic, thereby evading the protective barrier offered by LLINs and sustaining malaria transmission despite high LLIN coverage [80]. In a recent study (2018) in the Lake Region of Tanzania, An. funestus s.l. were the dominant malaria vector followed by An. arabiensis and An. gambiae s.s. where they all showed similar feeding patterns of feeding indoors and outdoors [37]. Current control measures for malaria (LLINs) and schistosomiasis (MDA) are not sufficient in reducing exposures to these diseases, further emphasizing the need for environmental control.

### Limitations

This study was subject to some limitations. We were unable to distinguish between current and past infection of schistosomiasis, which likely explains the findings of very high (94.3%) schistosomiasis seroprevalence in the study area. Children between five and fourteen were included in this study since Praziquantel tablets were not recommended for children under four years old (or under 94 cm in height) at the time of recruitment [18]. As of February 2022, children over the age of two could be treated with Praziquantel–if children between the ages of two and five were recruited in our study, we could have investigated a more discernible age-infection profile for schistosomiasis [19]. Previous research in communities along Lake Victoria found that children between the ages of one and five could demonstrate high schistosomiasis infection prevalence (i.e., 39.3% prevalence of intestinal schistosomiasis in an Ugandan community along Lake Victoria) and that IgG antibodies can persist for years after initial exposure [81–83]. To mitigate this challenge, we conducted a secondary analysis to assess the performance of the sRDT by measuring the sensitivity and specificity of the sRDT using ELISA kits as the diagnostic standard and the potential distinction between current and past infection, using the sRDT. The results from the ELISA kits were all negative–likely reflecting the methods used to elute Schistosoma antibodies from the mRDTs. The clear and distinct test bands distinguished by photos of the sRDT also likely reflect higher antibody titers (i.e., recent infection) and faint test band likely reflect lower antibody titers (i.e., past infection). In this analysis, we conducted a secondary analysis to investigate whether accounting for sRDTs with clear and distinct band (i.e., high antibody titers, recent infection), would influence the factors associated with schistosomiasis, but the results from the preliminary and secondary analysis were consistent, with no additional findings arising from the secondary analysis. We were also unable to distinguish between intestinal and urogenital schistosomiasis infections since the sRDT detects both *Schistosoma* species. This restricted our ability to evaluate the gradient of infection exposures to *S*. *mansoni* (intestinal schistosomiasis) near the lake and *S*. *haematobium* (urogenital schistosomiasis) inland [39, 81]. The gold standard diagnostic test for intestinal and urogenital schistosomiasis are both processed in a laboratory that require trained personnel and include a duplicate Kato-Katz from one stool sample, and urine filtration methods, respectively [22]. These two current methods do not meet the ASSURED (affordable, sensitive, specific, user-friendly, rapid, equipment-free, and delivered to those who need it) criteria for diagnostic tests since they are not easily administered at the village-level, where access to schistosomiasis diagnosis and treatment are needed [84]. Symptoms for malaria and schistosomiasis are non-specific which emphasize the need for adequate and accessible testing. Rapid diagnostic tests, such as the Schistosoma ICT IgG-IgM, have the potential to strengthen the health system by adequately diagnosing and providing the correct treatment for schistosomiasis [2, 22].

Another limitation to note is that the cross-sectional survey and community mapping were both administered and conducted during the dry season in January and August, respectively. Malaria transmission in Misungwi is year-round, which is indicative of the breeding site preference (i.e., permanent, or semi-permanent, medium-sized ponds with water most of the year) of *An*. *funestus*, the dominant *Anopheles* species in the study area. However, seasonality could play an important role for schistosomiasis transmission since there are many species of *Biomphalaria* and *Bulinus* snails, each with their own unique aquatic niche and breeding site preference (i.e., permanent lakes, temporary ponds) [85, 86]. Some *Biomphalaria* and *Bulinus* snails species can also aestivate and survive during the dry season, which increases the risk of schistosomiasis during the rainy season [87]. The dry season may also alter water-contact behaviors and increase the risk for schistosomiasis, which was noted in other longitudinal studies investigating risk factors for schistosomiasis but could not be assessed in this cross-sectional study [61]. This once again emphasizes the importance of effective environmental control and supplementary prevention strategies for malaria and schistosomiasis.

## Conclusion

The utilization of both qualitative and quantitative data allowed us to gain a comprehensive understanding of population vulnerabilities and socio-ecological factors associated with malaria, schistosomiasis, and co-infection among school-aged children in Misungwi, Lake Victoria Zone, Tanzania. To our knowledge, this is the first study to investigate individual- and household-level social determinants, and household-level environmental determinants associated with malaria, schistosomiasis, and co-infection using a mixed-method approach in an African setting. The geographic overlap and co-infection of malaria and schistosomiasis in Misungwi, Tanzania, suggest that integrated or supplementary control strategies are necessary to reduce disease transmission. Future studies can examine the coordination between stakeholders or funding sources for malaria and schistosomiasis control programs to assess the feasibility of integrating environmental control with current strategies. This study improves our understanding of social and environmental factors that are associated with malaria, schistosomiasis, and co-infection which can inform potential entry points for integrated disease prevention and control.

## Supporting information

S1 Text Sample size derivation. (DOCX)

S2 Text Variable definition. (DOCX)

S3 Text Inclusivity in global research. (DOCX)

S4 Text Univariable association. (DOCX)

S5 Text Six community maps. (DOCX)
