# Supplementary material for: Assessing risk factors for malaria and schistosomiasis among children in Misungwi, Tanzania, an area of co-endemicity: A mixed methods study
Source: PLOS Glob Public Health. 2023 Nov 22;3(11):e0002468. doi: 10.1371/journal.pgph.0002468 (PMC10664891; doi:10.1371/journal.pgph.0002468)
Supplement: S1 Text — (DOCX) [file pgph.0002468.s001.docx]

**S1 File:** Sample Size Derivation

The sample size for collection of questionnaire data is based on the number of children that will be targeted during the January 2022 cross-sectional survey as part of the Misgungwi Net Trial (50 children per cluster in 84 clusters; n=4300) and available funding (1,300 children maximum). In order to estimate the prevalence of past and recent schistosomiasis infection (as measured by IgG and IgM antibody detection, respectively) with 80% power and 5% precision, and to detect a difference of 30% and 50% in schistosomiasis IgM and IgG antibody prevalence between two groups for hypothesized risk factors (e.g. low and high knowledge scores), we need to use the sample size calculation for comparing two proportions for cluster randomized trials (equation 1). Equation 1 is a standard sample size calculation for two proportions but inflated by a factor of: (1+(m-1) $\rho$) to account for the independence violation in the outcome[1,2].

n= $\frac{\left( z_{\frac{\alpha}{2}}+z_{\beta} \right)^{2} \left[ P1\left( 1-P1 \right)+P2\left( 1-P2 \right) \right]\left[ 1+\left( m-1 \right)\rho\right]}{\left( P1-P2 \right)^{2}}$ (1)

Using the constraints and parameters of the study (that are displayed in equation 2), we can estimate the number of children required in each cluster: $z_{\alpha/2}$ _=_ 1.96 ($\alpha$ =0.05), $z_{\beta}$ _=_ 0.84 (80% power), P1 = 0.5 (hypothesized schistosomiasis prevalence in Tanzania), P2 = 0.3 (detect a 20% difference between high-risk (50%) and moderate-risk (10-50%) communities that have different recommended strategies for schistosomiasis prevention), n=650 (number of individuals in two groups with 1300 total tests), m = 15-50 (13 clusters of 50 children [m] or 42 clusters with 15 children[m])[3]:

650= $\frac{\left( 1.96+0.84 \right)^{2} [0.5\left( 0.5 \right)+0.3\left( 0.7 \right)][1+\left( m-1 \right)\rho]}{\left( 0.5-0.3 \right)^{2}}$ (2)

The intra cluster correlation ($\rho$) for the schistosomiasis prevalence within the clusters in the study is not known. We do however know that the value of $\rho$ is between 0 (independence of schistosomiasis prevalence among children in the cluster) and 1 (total dependence of schistosomiasis prevalence among children of the clusters) and that the number of children per cluster can range between 15-50 (based on the number of households being targeted). We can then solve for the number of children per cluster for this study using a range of $\rho$and m that make right side of equation 2 as close to 650 children per group without going over.

For m= 15, the intra cluster correlation is assumed to be 0.44 with 646 children per group; and when m = 50, the intracluster correlation is assumed to be 0.12 with 620 children per group. Accounting for increasing power with an increasing number of clusters and to conservatively select an intra cluster correlation above 0.2, we can then look at the baseline cross-sectional study from this trial to determine the number of children who would be eligible for praziquantel tablets during MDA (children greater than or equal to 5) [1]. In Fig 1, we can see that the number of children eligible for MDA in the baseline cross-sectional study varied by cluster, for a total of 3023 children from 2122 houses – which is over the limit of 1300 rapid diagnostic tests.

**Fig 1:** Number of children 5-14 years old selected in the baseline cross-sectional survey in 2018. Map content was produced with Esri ArcGIS software using study data and data provided by GADM available online: <https://gadm.org/download_country.html>.


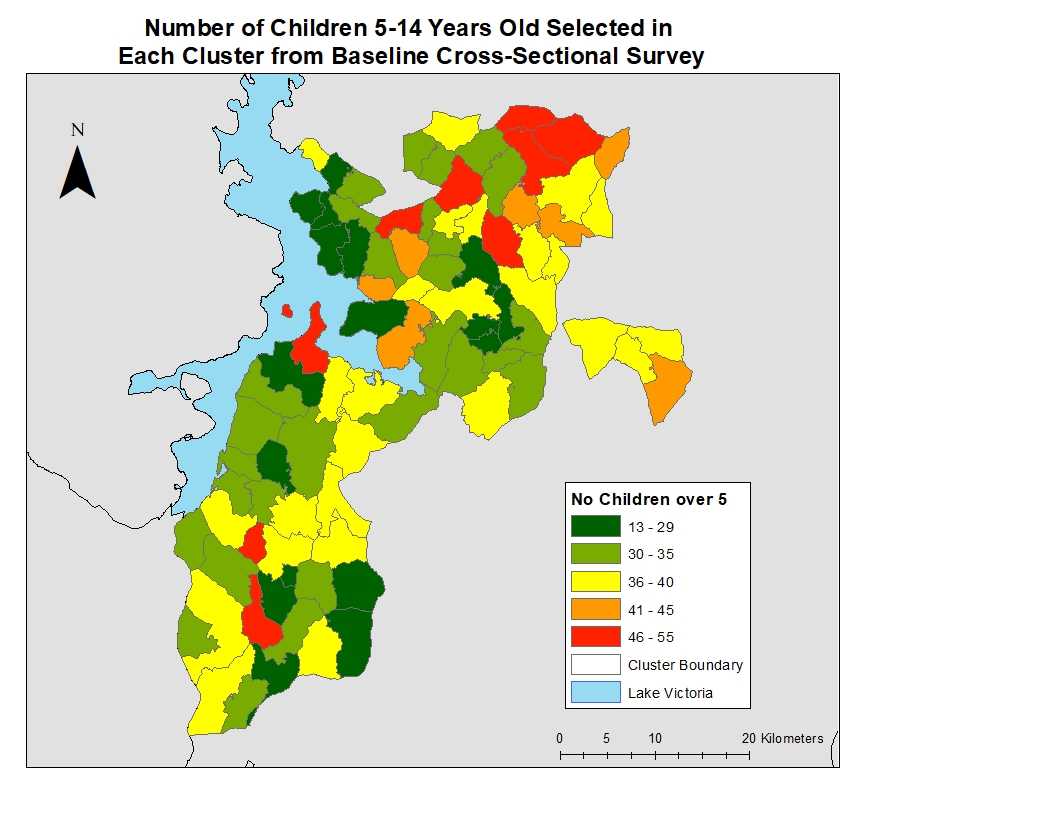


The main goal of this chapter is to understand factors that are independently and jointly associated with malaria and schistosomiasis. One objective is to report the prevalence of malaria, schistosomiasis, and co-infection and another objective is to assess determinants that are associated with malaria and schistosomiasis. We should target clusters that have a higher percentage of malaria prevalence to ensure that we are examining clusters with children that are infected with both malaria and schistosomiasis. However, we should not restrict our sample to only clusters with a high prevalence to ensure variability in the results of the 36-months cross-sectional study in January 2022.

Fig 2A outlines the percent of children eligible for MDA (5 years old or greater) in each cluster, that were tested for malaria using a rapid diagnostic test (RDT), and who tested positive for malaria. The distribution of the prevalence of infection as measured by RDT is skewed (as seen in figure 3), with a median cluster prevalence of 53% and a minimum and maximum cluster prevalence of 0% and 89%, respectively. By restricting the sample to clusters with a prevalence of malaria infection greater than 40% (as seen in Fig 2B), the sample is reduced to 2180 children from 1519 households. For ease of sampling, we can select one child per household to be tested for schistosomiasis, but that would still be an excess of 219 children. Returning to Fig 2A and 2B, we can visually identify two areas of higher malaria prevalence in yellow, orange, and red (prevalence of malaria between 41% and 89%) - one in the southern region and one in the northern region along Lake Victoria. By geographically selecting clusters in these areas (as seen in Fig 2C), we can reduce the sample to 48 clusters with 1612 children from 1140 households. We removed cluster 22 and 69 from the sample because: 1) cluster 22, which is in the northern region along lake Victoria, did not meet the eligibility requirement for the ongoing bed-net trial and has been excluded from the study and 2) cluster 69, a smaller cluster in the southern region, is comprised of a small number of households (i.e. 11 households sampled at baseline and 13 households sampled in a recent cross-sectional survey). Returning to equation 3 - with 48 clusters (cluster number 15-16,18-21,23,25-26,31-33,37,48,50-51,54-68, 70-86; illustrated in figure 4), there is a sufficient number of schistosomiasis RDTs to select 27 children (one per household) per cluster with an intra cluster correlation of 0.23 for a total of 648 children in each group.

**Figure 2:** Percent of Children 5-14 years old with positive RDTs in selected clusters from the baseline cross-sectional survey in 2018. Map content was produced with Esri ArcGIS software using study data and data provided by GADM available online: <https://gadm.org/download_country.html>.


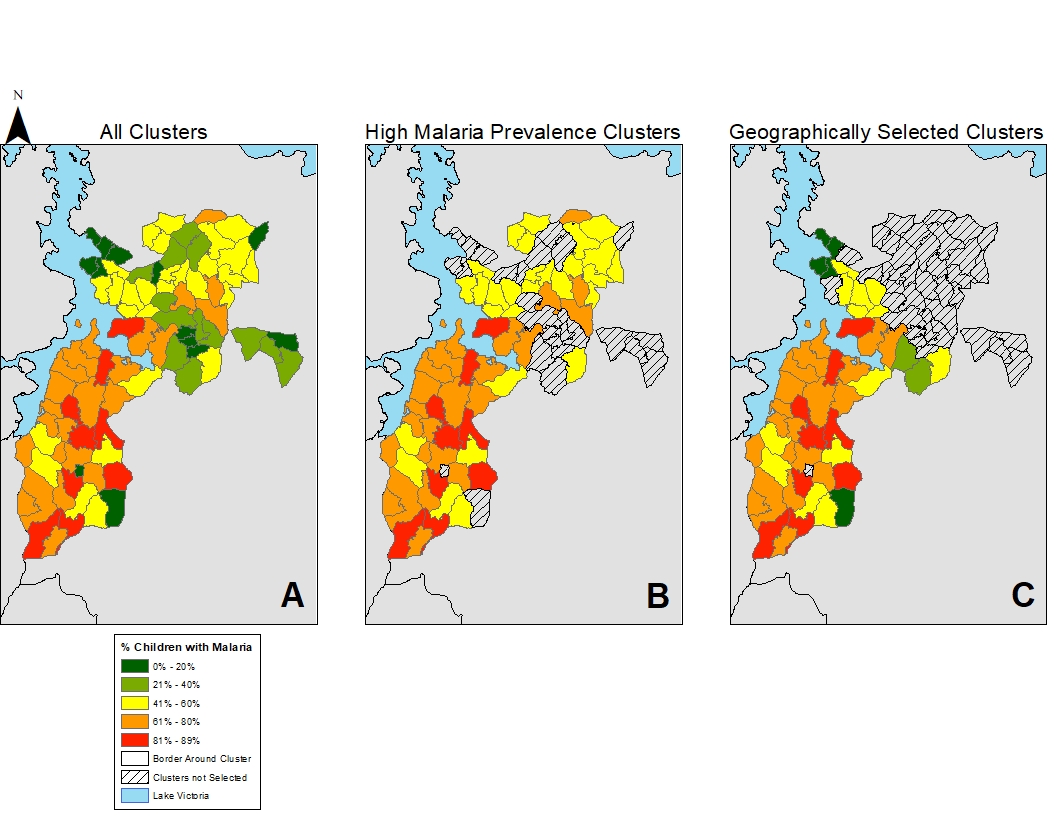

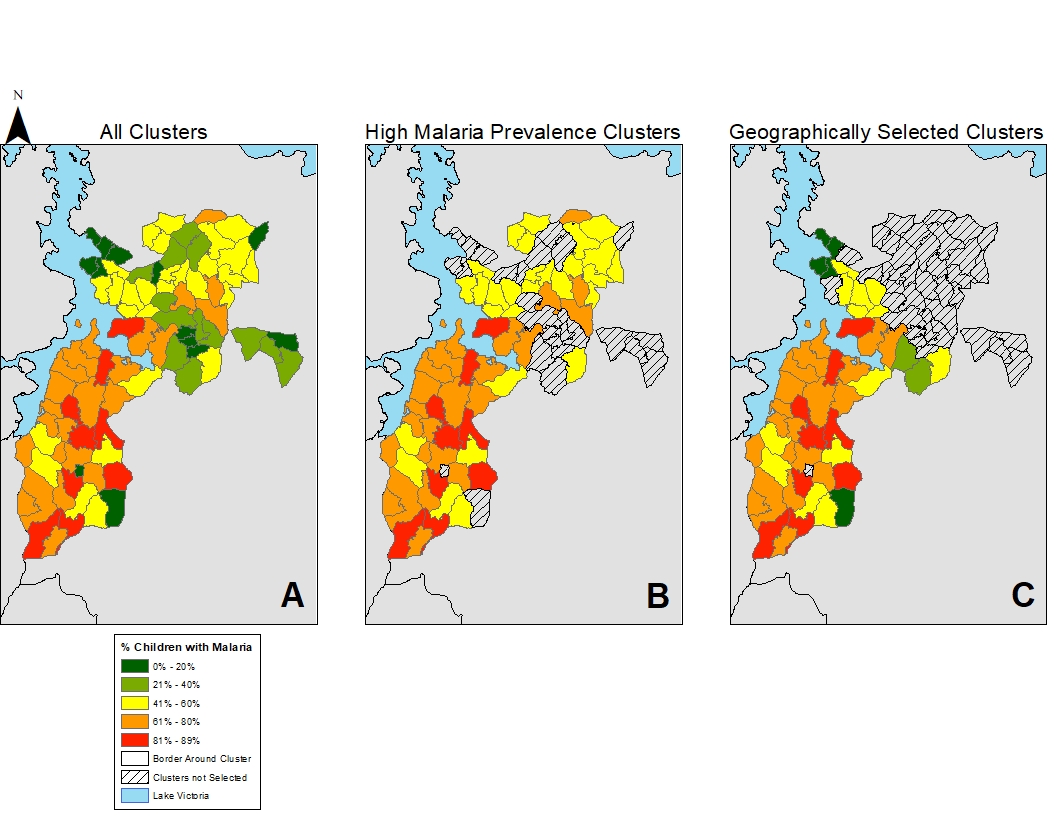


**Figure 3:** Distribution of prevalence of malaria infection as measured by RDT in the baseline cross-sectional survey in 2018. Map content was produced with Esri ArcGIS software using study data and data provided by GADM available online: <https://gadm.org/download_country.html>.


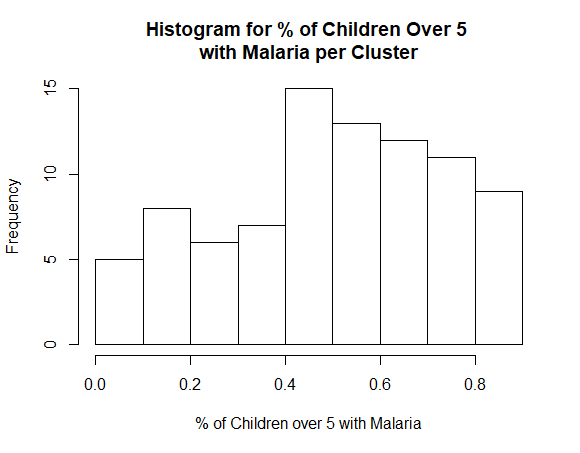


**Figure 4:** Clusters Selected for Schistosomiasis Rapid Diagnostic Test in 36-Month Cross-Sectional Survey (one child ≥5 years old per household). Map content was produced with Esri ArcGIS software using study data and data provided by GADM available online: <https://gadm.org/download_country.html>.


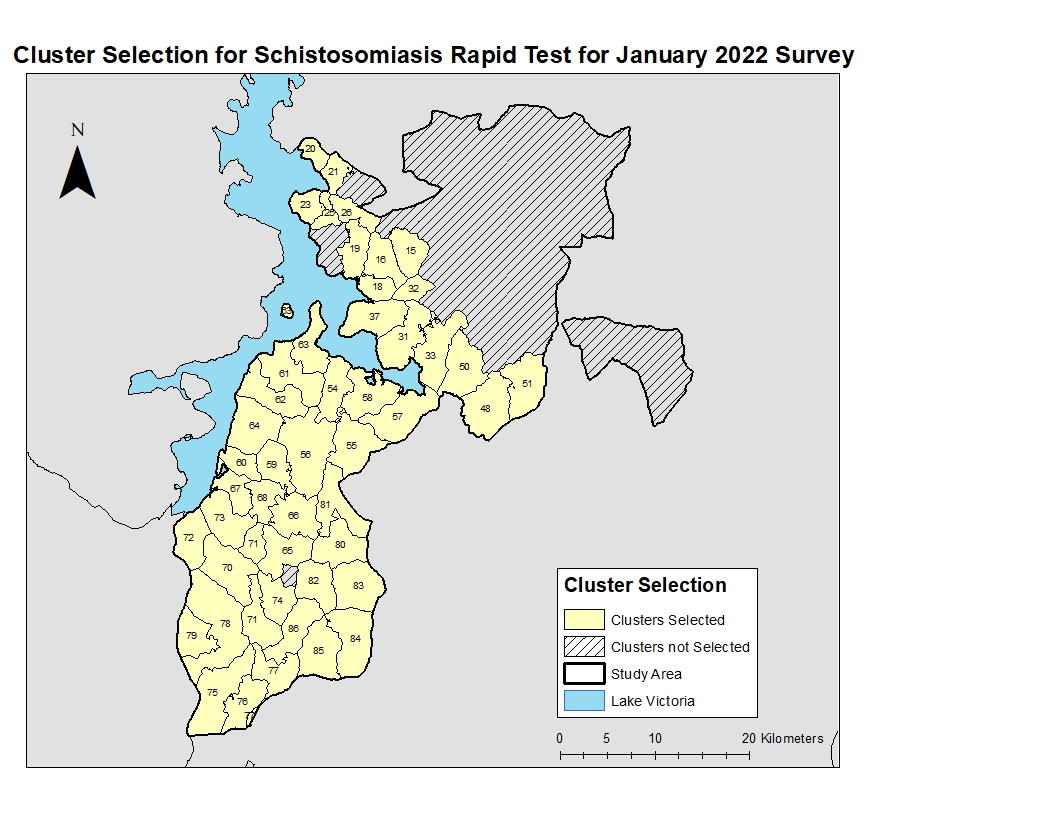


**References:**

1. Campbell MK, Thomson S, Ramsay CR, MacLennan GS, Grimshaw JM. Sample size calculator for cluster randomized trials. Comput Biol Med. 2004;34: 113–125. doi:10.1016/S0010-4825(03)00039-8

2. Eldridge S, Kerry SM. A practical guide to cluster randomised trials in health services research. Chichester, West Sussex, U.K: John Wiley & Sons; 2012.

3. Crompton DWT, World Health Organization. Preventive chemotherapy in human helminthiasis : coordinated use of anthelminthic drugs in control interventions : a manual for health professionals and programme managers. Chim Helminthiases Chez Homme Util Coord Médicam Anthelminthiques Pour Interv Lutte Man À Intent Prof Santé Adm Programme. 2006; 62.
