# Supplementary material for: Assessing risk factors for malaria and schistosomiasis among children in Misungwi, Tanzania, an area of co-endemicity: A mixed methods study
Source: PLOS Glob Public Health. 2023 Nov 22;3(11):e0002468. doi: 10.1371/journal.pgph.0002468 (PMC10664891; doi:10.1371/journal.pgph.0002468)
Supplement: S2 Text — (DOCX) [file pgph.0002468.s002.docx]

**Variable Definitions**

| **Variable** | **Variable Definition** |
| --- | --- |
| **Social** **determinant (individual-level)** | |
| Age | Age of selected child |
| Sex | Sex of selected child |
| **Social determinant (household-level)** | |
| *Malaria and schistosomiasis* | |
| Socioeconomic status | Wealth score as a proxy for household socioeconomic status using a Principal Component Analysis of household assets and dwelling characteristics. [1,2] |
| Education | Head of household education |
| Occupation | Head of household occupation |
| *Malaria only* | |
| Knowledge of disease | Head of household identifies at least once correct malaria symptoms (fever, nausea, diarrhea, loss of energy, pain) and identifies mosquito bites as mechanism of transmission of malaria. [3,4] |
| Perception of number of people with disease in village | Perception of the disease was measured for the head of household by the question “in your opinion, how many people in your village have malaria?” with the following options: none, few, some, many, everyone, don’t know (was not read out as an option). |
| Concern of disease personally | Concern of the disease was measured for the head of household by the question “are you concerned about malaria personally?” with the following options: not at all concerned, slightly concerned, somewhat concerned, moderately concerned, extremely concerned, don’t know (was not read out as an option). |
| LLIN ownership | Proportion of households with at least one LLIN [5,6] |
| LLIN access | Proportion of population that could sleep under a LLIN if each LLIN in the household were used by 2 people [6] |
| *Schistosomiasis only* | |
| Knowledge of disease | Head of household identifies at least once correct schistosomiasis symptoms (blood in diarrhea, blood in urine, swelling of belly, stomach-ache, vomiting, infertility, no symptoms) and identifies at least once correct schistosomiasis mode of transmission (entering river, entering lake, poor hygiene). [3,4] |
| Perception of number of people with disease in village | Perception of the disease was measured for the head of household by the question “in your opinion, how many people in your village have schistosomiasis?” with the following options: none, few, some, many, everyone, don’t know (was not read out as an option). |
| Concern of disease personally | Concern of the disease was measured for the head of household by the question “are you concerned about schistosomiasis personally?” with the following options: not at all concerned, slightly concerned, somewhat concerned, moderately concerned, extremely concerned, don’t know (was not read out as an option). |
| Improved drinking Water Source | The proportion of the population that uses a water source that delivers safe water (piped water and dug wells). [7] |
| Improved Sanitation Facility | The proportion of the population that uses improved sanitation facilities (flush toilet, ventilated improved pit latrines). [7] |
| Improved Hygiene | The proportion of the population that uses a handwashing facility (sink in dwelling, sink in yar, bucket/jug/kettle) with soap and water. [7] |
| **Environmental determinant** | |
| Temperature | Retrieved for each household. Daytime LST from January 1-February 1, 2022, at a 1km spatial resolution, scaled by a factor 0.02 and converted from Kelvin to Celsius (-273.15). Gridded raster data retrieved from Moderate Resolution Imaging Spectroradiometer (MODIS). [8] |
| Precipitation | Retrieved for each household. Precipitation average (1970-2000) for January and February at a 1km spatial resolution. Gridded raster data retrieved from WorldClim. [9] |
| NDVI | Retrieved for each household. Normalized Vegetation index for January 2022, at a 250 m spatial resolution, scaled by a factor of 0.0001. Gridded raster data retrieved from MODIS. [10] |
| Population density (people/km^2^) | Retrieved for each household. Population density defined as the number of 100 people per kilometer^2^ in 2020 at a 1km spatial resolution. Gridded raster data retrieved from WorldPop. [11] |
| Distance to Lake Victoria | Retrieved for each household. The Euclidean distance to Lake Victoria calculated using GIS ArcMap version 10.8.1 (ESRI, Redlands, CA, USA). [12] |

**References**

1. Vyas S, Kumaranayake L. Constructing socio-economic status indices: how to use principal components analysis. Health Policy Plan. 2006;21: 459–468. doi:10.1093/heapol/czl029

2. Homenauth E, Kajeguka D, Kulkarni MA. Principal component analysis of socioeconomic factors and their association with malaria and arbovirus risk in Tanzania: a sensitivity analysis. J Epidemiol Community Health. 2017; jech-2017-209119. doi:10.1136/jech-2017-209119

3. Parisi S, Mazigo HD, Kreibich S, Puchner K, Kasang C, Mueller A. Factors associated with relevant knowledge of intestinal schistosomiasis and intention to participate in treatment campaigns: a cross sectional survey among school children at Ijinga Island on Lake Victoria, North-Western Tanzania. BMC Public Health. 2019;19: 1762. doi:10.1186/s12889-019-8091-4

4. Muhumuza S, Olsen A, Katahoire A, Nuwaha F. Uptake of Preventive Treatment for Intestinal Schistosomiasis among School Children in Jinja District, Uganda: A Cross Sectional Study. Speybroeck N, editor. PLoS ONE. 2013;8: e63438. doi:10.1371/journal.pone.0063438

5. By-Nc-Sa C. World malaria report 2022. 2022; 372.

6. WHO Guidelines for malaria. [cited 13 Dec 2022]. Available: https://www.who.int/publications-detail-redirect/guidelines-for-malaria

7. JMP METHODOLOGY 2017 UPDATE & SDG BASELINES. MBE. 2016;13: 2–2. doi:10.3934/mbe.2017024

8. Wan, Zhengming, Hook, Simon, Hulley, Glynn. MOD11A2 MODIS/Terra Land Surface Temperature/Emissivity 8-Day L3 Global 1km SIN Grid V006. NASA EOSDIS Land Processes DAAC; 2015. doi:10.5067/MODIS/MOD11A2.006

9. Fick SE, Hijmans RJ. WorldClim 2: new 1‐km spatial resolution climate surfaces for global land areas. Int J Climatol. 2017;37: 4302–4315. doi:10.1002/joc.5086

10. Didan, Kamel. MOD13Q1 MODIS/Terra Vegetation Indices 16-Day L3 Global 250m SIN Grid V006. NASA EOSDIS Land Processes DAAC; 2015. doi:10.5067/MODIS/MOD13Q1.006

11. WorldPop,, Bondarenko, Maksym. Individual Countries 1km Population Density (2000-2020). University of Southampton; 2020. doi:10.5258/SOTON/WP00674

12. Natural Earth » 1:10m Physical Vectors - Free vector and raster map data at 1:10m, 1:50m, and 1:110m scales. [cited 24 Feb 2023]. Available: https://www.naturalearthdata.com/downloads/10m-physical-vectors/
