# Supplementary material for: Assessing risk factors for malaria and schistosomiasis among children in Misungwi, Tanzania, an area of co-endemicity: A mixed methods study
Source: PLOS Glob Public Health. 2023 Nov 22;3(11):e0002468. doi: 10.1371/journal.pgph.0002468 (PMC10664891; doi:10.1371/journal.pgph.0002468)
Supplement: S4 Text — (DOCX) [file pgph.0002468.s004.docx]

**Supplemental Table 1**: Results of primary mixed effects logistic regression analysis of factors associated with malaria, schistosomiasis, and co-infection (n = 1122)

|  | | **Malaria** | | | **Schistosomiasis** | | | **Co-infection** | | |
| --- | --- | --- | --- | --- | --- | --- | --- | --- | --- | --- |
|  |  | OR* | (95% CI) | P-value | OR* | (95% CI) | P-value | OR* | (95% CI) | P-value |
| Individual determinants (child) | | | | | | | | | | |
| Age of Selected Child | | | | | | | | | | |
|  | | 1.16 | (1.11-1.22) | <0.0001 | 1.23 | (1.10-1.37) | 0.0002 | 1.17 | (1.11-1.23) | <0.0001 |
| Sex of Selected Child | | | | | | | | | | |
|  | Girl | REF | - | - | REF | - | - | REF | - | - |
|  | Boy | 1.44 | (1.12-1.86) | 0.0050 | 1.65 | (0.94-2.91) | 0.0785 | 1.52 | (1.18-1.97) | 0.0012 |
| Social determinants (household) | | | | | | | | | | |
| Socioeconomic status | | | | | | | | | | |
|  | Lowest | 1.83 | (1.19-2.81) | 0.0053 | 1.32 | (0.51-3.35) | 0.5615 | 1.78 | (1.16-2.73) | 0.6784 |
|  | Low | 1.92 | (1.12-2.92) | 0.0025 | 0.85 | (0.35-2.05) | 0.7288 | 1.92 | (1.26-2.93) | 0.1413 |
|  | Average | 1.39 | (0.92-2.12) | 0.1203 | 1.06 | (0.42-2.65) | 0.8917 | 1.37 | (0.90-2.01) | 0.0024 |
|  | High | 1.13 | (0.72-1.76) | 0.5804 | 0.80 | (0.32-1.97) | 0.6349 | 1.09 | (0.70-1.71) | 0.0078 |
|  | Highest | REF | - | - | REF | - | - | REF | - | - |
| Head of household education | | | | | | | | | | |
|  | None | 1.34 | (0.67-2.66) | 0.4041 | 0.73 | (0.18-2.90) | 0.6556 | 1.35 | (0.68-2.70) | 0.3862 |
|  | Primary | 1.10 | (0.56-2.12) | 0.7798 | 0.85 | (0.22-3.23) | 0.8207 | 1.12 | (0.57-2.18) | 0.7316 |
|  | Secondary or higher | REF | - | - | REF | - | - | REF | - | - |
| Knowledge of disease (malaria) | | | | | | | | | | |
|  | Yes | 0.82 | (0.60-1.12) | 0.2173 | 0.67 | (0.32-1.39) | 0.2902 | 0.82 | (0.60-1.12) | 0.2270 |
|  | No | REF | - | - | REF | - | - | REF | - | - |
| Perception of disease (malaria) | | | | | | | | | | |
|  | None | 1.44 | (0.52-3.96) | 0.4797 | 0.47 | (0.08-2.75) | 0.4029 | 1.62 | (0.59-4.46) | 0.3470 |
|  | Few/some | 1.11 | (0.82-1.51) | 0.4866 | 1.49 | (0.76-2.93) | 0.2435 | 1.17 | (0.86-1.59) | 0.3146 |
|  | Many/everyone | REF | - | - | REF | - | - | REF | - | - |
|  | Don’t know | 1.97 | (0.93-4.18) | 0.0737 | 1.97 | (0.93-4.18) | 0.0737 | 1.67 | (1.21-2.30) | 0.0018 |
| Concern of disease personally (malaria) | | | | | | | | | | |
|  | Not at all concerned | 0.92 | (0.66-1.26) | 0.6024 | 1.75 | (0.80-3.83) | 0.1609 | 0.97 | (0.70-1.34) | 0.8821 |
|  | Somewhat to slightly concerned | 0.94 | (0.66-1.34) | 0.7472 | 1.89 | (0.74-4.79) | 0.1783 | 0.92 | (0.64-1.31) | 0.6565 |
|  | Moderately to extremely concerned | REF | - | - | REF | - | - | REF | - | - |
|  | Don’t know | 1.55 | (0.67-3.40) | 0.3183 | 0.34 | (0.10-1.19) | 0.0927 | 1.23 | (0.54-2.78) | 0.6157 |
| LLIN Access | | | | | | | | | | |
|  | Yes | REF | - | - | REF | - | - | REF | - | - |
|  | No | 1.64 | (1.22-2.21) | 0.0011 | 1.76 | (0.98-3.17) | 0.0579 | 1.62 | (1.20-2.18) | 0.0014 |
| Knowledge of disease (schistosomiasis) | | | | | | | | | | |
|  | Yes | 0.71 | (0.53-0.93) | 0.0120 | 0.86 | (0.48-1.53) | 0.6137 | 0.71 | (0.54-0.94) | 0.0161 |
|  | No | REF | - | - | REF | - | - | REF | - | - |
| Perception of disease (schistosomiasis) | | | | | | | | | | |
|  | None | 0.94 | (0.52-1.71) | 0.8634 | 1.52 | (0.53-4.34) | 0.4325 | 1.17 | (0.64-2.13) | 0.6082 |
|  | Few/some | 1.32 | (0.85-2.02) | 0.2103 | 1.51 | (0.68-3.31) | 0.3068 | 1.54 | (0.99-2.40) | 0.0539 |
|  | Many/everyone | REF | - | - | REF | - | - | REF | - | - |
|  | Don’t know | 1.73 | (1.13-2.66) | 0.0116 | 2.49 | (1.10-5.57) | 0.0279 | 2.18 | (1.41-3.39) | 0.0005 |
| Concern of disease personally (schistosomiasis) | | | | | | | | | | |
|  | Not at all concerned | 0.73 | (0.54-0.98) | 0.0411 | 1.39 | (0.72-2.67) | 0.3239 | 0.80 | (0.59-1.07) | 0.1413 |
|  | Somewhat to slightly concerned | 0.75 | (0.52-1.09) | 0.1336 | 0.99 | (0.47-2.11) | 0.9963 | 0.66 | (0.46-0.96) | 0.0320 |
|  | Moderately to extremely concerned | REF | - | - | REF | - | - | REF | - | - |
|  | Don’t know | 0.78 | (0.43-1.41) | 0.4181 | 1.59 | (0.41-6.14) | 0.4985 | 0.84 | (0.47-1.50) | 0.5627 |
| Drinking water | | | | | | | | | | |
|  | Improved | REF | - | - | REF | - | - | REF | - | - |
|  | Unimproved | 2.08 | (1.42-3.07) | 0.0002 | 1.01 | (0.41-2.43) | 0.9871 | 2.02 | (1.38-2.98) | 0.0003 |
| Sanitation facility | | | | | | | | | | |
|  | Improved | REF | - | - | REF | - | - | REF | - | - |
|  | Pit latrine | 1.81 | (1.18-2.73) | 0.0059 | 1.18 | (0.52-2.67) | 0.6806 | 1.83 | (1.20-2.79) | 0.0049 |
|  | Bush Toilet | 2.00 | (1.21-3.29) | 0.0063 | 1.11 | (0.39-3.16) | 0.8359 | 1.92 | (1.16-3.17) | 0.0105 |
| Hygiene | | | | | | | | | | |
|  | Improved | REF | - | - | REF | - | - | REF | - | - |
|  | Unimproved | 1.21 | (0.92-1.60) | 0.1621 | 1.09 | (0.60-1.98) | 0.7644 | 1.27 | (0.97-1.63) | 0.0796 |
| Environmental determinants | | | | | | | | | | |
| Temperature (°C) | | | | | | | | | | |
|  |  | 1.07 | (0.98-1.18) | 0.1239 | 0.98 | (0.79-1.21) | 0.8717 | 1.06 | (0.97-1.17) | 0.1475 |
| Precipitation (mm) | | | | | | | | | | |
|  |  | 0.94 | (0.85-1.04) | 0.2443 | 1.04 | (0.82-1.30) | 0.7638 | 0.94 | (0.86-1.04) | 0.2809 |
| NDVI | | | | | | | | | | |
|  | Sparse vegetation | 1.13 | (0.76-1.68) | 0.5437 | 1.37 | (0.69-3.15) | 0.4560 | 1.13 | (0.76-1.69) | 0.5223 |
|  | Dense vegetation | REF | - | - | REF | - | - | REF | - | - |
| Population density | | | | | | | | | | |
|  | <100 per km^2^ | 3.02 | (1.76-5.17) | <0.0001 | 1.43 | (0.44-4.66) | 0.5478 | 2.79 | (1.66-4.69) | 0.0001 |
|  | 100-200 per km^2^ | 2.34 | (1.64-3.33) | <0.0001 | 1.35 | (0.57-3.15) | 0.4877 | 2.38 | (1.68-3.36) | <0.0001 |
|  | >200 per km^2^ | REF | - | - | REF | - | - | REF | - | - |
| Distance to Lake Victoria (km) | | | | | | | | | | |
|  | Near (<1km) | REF | - | - | REF | - | - | REF | - | - |
|  | Middle | 1.18 | (0.66-2.09) | 0.5656 | 1.11 | (0.38-3.25) | 0.8397 | 1.12 | (0.63-1.98) | 0.6962 |
|  | Far (>5km) | 2.54 | (1.36-4.75) | 0.0034 | 1.39 | (0.33-5.84) | 0.6526 | 2.37 | (1.29-4.35) | 0.0051 |
| * Random effects for cluster and adjusted for intervention arm  **Abbreviations:** OR: Odds Ratio; LLIN: Long Lasting Insecticidal Nets; NDVI: Normalized Difference Vegetation Index | | | | | | | | | | |

**Supplemental Table 2**: Results of secondary mixed effects logistic regression analysis of factors associated with malaria infection, strong-positive schistosomiasis seropositivity, and malaria and strong-positive schistosomiasis co-infection (n = 1122)

|  | | **Malaria** | | | **Schistosomiasis** | | | **Co-infection** | | |
| --- | --- | --- | --- | --- | --- | --- | --- | --- | --- | --- |
|  |  | OR* | (95% CI) | P-value | OR* | (95% CI) | P-value | OR* | (95% CI) | P-value |
| Individual determinants (child) | | | | | | | | | | |
| Age of Selected Child | | | | | | | | | | |
|  | | 1.16 | (1.11-1.22) | <0.0001 | 1.18 | (1.12-1.24) | <0.0001 | 1.24 | (1.15-1.33) | <0.0001 |
| Sex of Selected Child | | | | | | | | | | |
|  | Girl | REF | - | - | REF | - | - | REF | - | - |
|  | Boy | 1.44 | (1.12-1.86) | 0.0050 | 2.40 | (1.81-3.18) | <0.0001 | 2.45 | (1.70-3.53) | <0.0001 |
| Social determinants (household) | | | | | | | | | | |
| Socioeconomic status | | | | | | | | | | |
|  | Lowest | 1.83 | (1.19-2.81) | 0.0053 | 0.87 | (0.56-1.36) | 0.3488 | 1.43 | (0.82-2.52) | 0.7543 |
|  | Low | 1.92 | (1.12-2.92) | 0.0025 | 1.17 | (0.76-1.80) | 0.4122 | 1.32 | (0.75-2.32) | 0.6988 |
|  | Average | 1.39 | (0.92-2.12) | 0.1203 | 0.83 | (0.54-1.28) | 0.4548 | 1.12 | (0.63-1.98) | 0.3239 |
|  | High | 1.13 | (0.72-1.76) | 0.5804 | 0.80 | (0.51-1.26) | 0.5520 | 0.90 | (0.49-1.67) | 0.2089 |
|  | Highest | REF | - | - | REF | - | - | REF | - | - |
| Head of household education | | | | | | | | | | |
|  | None | 1.34 | (0.67-2.66) | 0.4041 | 1.13 | (0.54-2.37) | 0.7401 | 1.60 | (0.53-4.83) | 0.3957 |
|  | Primary | 1.10 | (0.56-2.12) | 0.7798 | 1.15 | (0.56-2.35) | 0.6902 | 1.72 | (0.60-5.03) | 0.3088 |
|  | Secondary or higher | REF | - | - | REF | - | - | REF | - | - |
| Knowledge of disease (malaria) | | | | | | | | | | |
|  | Yes | 0.82 | (0.60-1.12) | 0.2173 | 0.65 | (0.47-0.90) | 0.0097 | 0.69 | (0.46-1.03) | 0.0723 |
|  | No | REF | - | - | REF | - | - | REF | - | - |
| Perception of disease (malaria) | | | | | | | | | | |
|  | None | 1.44 | (0.52-3.96) | 0.4797 | 1.40 | (0.48-4.08) | 0.5277 | 2.22 | (0.67-7.32) | 0.1870 |
|  | Few/some | 1.11 | (0.82-1.51) | 0.4866 | 1.66 | (1.19-2.30) | 0.0023 | 1.45 | (0.95-2.21) | 0.0832 |
|  | Many/everyone | REF | - | - | REF | - | - | REF | - | - |
|  | Don’t know | 1.97 | (0.93-4.18) | 0.0737 | 1.53 | (1.09-2.17) | 0.0143 | 1.56 | (1.01-2.41) | 0.0440 |
| Concern of disease personally (malaria) | | | | | | | | | | |
|  | Not at all concerned | 0.92 | (0.66-1.26) | 0.6024 | 1.10 | (0.78-1.55) | 0.5771 | 1.01 | (0.66-1.56) | 0.9398 |
|  | Somewhat to slightly concerned | 0.94 | (0.66-1.34) | 0.7472 | 1.06 | (0.73-1.55) | 0.7372 | 0.91 | (0.56-1.49) | 0.7216 |
|  | Moderately to extremely concerned | REF | - | - | REF | - | - | REF | - | - |
|  | Don’t know | 1.55 | (0.67-3.40) | 0.3183 | 2.06 | (0.92-4.58) | 0.0758 | 2.19 | (0.87-5.46) | 0.0929 |
| LLIN Access | | | | | | | | | | |
|  | Yes | REF | - | - | REF | - | - | REF | - | - |
|  | No | 1.64 | (1.22-2.21) | 0.0011 | 1.30 | (0.95-1.78) | 0.0963 | 1.79 | (1.16-2.76) | 0.0079 |
| Knowledge of disease (schistosomiasis) | | | | | | | | | | |
|  | Yes | 0.71 | (0.53-0.93) | 0.0120 | 1.18 | (0.89-1.57) | 0.2394 | 0.85 | (0.59-1.23) | 0.3975 |
|  | No | REF | - | - | REF | - | - | REF | - | - |
| Perception of disease (schistosomiasis) | | | | | | | | | | |
|  | None | 0.94 | (0.52-1.71) | 0.8634 | 1.14 | (0.60-2.13) | 0.6790 | 1.43 | (0.53-3.81) | 0.4701 |
|  | Few/some | 1.32 | (0.85-2.02) | 0.2103 | 1.48 | (0.91-2.31) | 0.1093 | 2.77 | (1.33-5.78) | 0.0064 |
|  | Many/everyone | REF | - | - | REF | - | - | REF | - | - |
|  | Don’t know | 1.73 | (1.13-2.66) | 0.0116 | 1.38 | (0.87-2.19) | 0.1665 | 2.62 | (1.25-5.54) | 0.0102 |
| Concern of disease personally (schistosomiasis) | | | | | | | | | | |
|  | Not at all concerned | 0.73 | (0.54-0.98) | 0.0411 | 0.83 | (0.61-1.14) | 0.2675 | 0.71 | (0.48-1.06) | 0.0974 |
|  | Somewhat to slightly concerned | 0.75 | (0.52-1.09) | 0.1336 | 0.94 | (0.64-1.38) | 0.7752 | 0.57 | (0.33-0.97) | 0.0385 |
|  | Moderately to extremely concerned | REF | - | - | REF | - | - | REF | - | - |
|  | Don’t know | 0.78 | (0.43-1.41) | 0.4181 | 0.83 | (0.44-1.57) | 0.5705 | 0.70 | (0.31-1.56) | 0.3863 |
| Drinking water | | | | | | | | | | |
|  | Improved | REF | - | - | REF | - | - | REF | - | - |
|  | Unimproved | 2.08 | (1.42-3.07) | 0.0002 | 1.17 | (0.79-1.74) | 0.4241 | 1.90 | (1.12-3.23) | 0.0175 |
| Sanitation facility | | | | | | | | | | |
|  | Improved | REF | - | - | REF | - | - | REF | - | - |
|  | Pit latrine | 1.81 | (1.18-2.73) | 0.0059 | 1.30 | (0.86-1.98) | 0.2015 | 1.87 | (1.01-3.48) | 0.0465 |
|  | Bush Toilet | 2.00 | (1.21-3.29) | 0.0063 | 1.38 | (0.83-2.28) | 0.2063 | 2.42 | (1.21-4.86) | 0.0125 |
| Hygiene | | | | | | | | | | |
|  | Improved | REF | - | - | REF | - | - | REF | - | - |
|  | Unimproved | 1.21 | (0.92-1.60) | 0.1621 | 0.99 | (0.73-1.33) | 0.9464 | 1.11 | (0.77-1.62) | 0.5582 |
| Environmental determinants | | | | | | | | | | |
| Temperature (°C) | | | | | | | | | | |
|  |  | 1.07 | (0.98-1.18) | 0.1239 | 0.92 | (0.84-1.01) | 0.1137 | 1.05 | (0.94-1.17) | 0.3491 |
| Precipitation (mm) | | | | | | | | | | |
|  |  | 0.94 | (0.85-1.04) | 0.2443 | 0.96 | (0.87-1.06) | 0.4835 | 0.91 | (0.81-1.02) | 0.1106 |
| NDVI | | | | | | | | | | |
|  | Sparse vegetation | 1.13 | (0.76-1.68) | 0.5437 | 1.13 | (0.74-1.74) | 0.5570 | 1.78 | (0.96-3.30) | 0.0652 |
|  | Dense vegetation | REF | - | - | REF | - | - | REF | - | - |
| Population density | | | | | | | | | | |
|  | <100 per km^2^ | 3.02 | (1.76-5.17) | <0.0001 | 1.02 | (0.55-1.88) | 0.9379 | 1.87 | (0.93-3.77) | 0.0775 |
|  | 100-200 per km^2^ | 2.34 | (1.64-3.33) | <0.0001 | 1.53 | (1.05-2.24) | 0.0272 | 2.69 | (1.67-4.33) | <0.0001 |
|  | >200 per km^2^ | REF | - | - | REF | - | - | REF | - | - |
| Distance to Lake Victoria (km) | | | | | | | | | | |
|  | Near (<1km) | REF | - | - | REF | - | - | REF | - | - |
|  | Middle | 1.18 | (0.66-2.09) | 0.5656 | 0.62 | (0.35-1.06) | 0.0949 | 1.00 | (0.48-2.11) | 0.9837 |
|  | Far (>5km) | 2.54 | (1.36-4.75) | 0.0034 | 0.46 | (0.25-0.85) | 0.0136 | 1.25 | (0.59-2.63) | 0.5519 |
| * Random effects for cluster and adjusted for intervention arm  **Abbreviations:** OR: Odds Ratio; LLIN: Long Lasting Insecticidal Nets; NDVI: Normalized Difference Vegetation Index | | | | | | | | | | |
