# Supplementary material for: Assessing risk factors for malaria and schistosomiasis among children in Misungwi, Tanzania, an area of co-endemicity: A mixed methods study
Source: PLOS Glob Public Health. 2023 Nov 22;3(11):e0002468. doi: 10.1371/journal.pgph.0002468 (PMC10664891; doi:10.1371/journal.pgph.0002468)
Supplement: S5 Text — (DOCX) [file pgph.0002468.s005.docx]

**Six Community Maps**

| **Gukwa, male** | **Gukwa, female** |
| --- | --- |
| **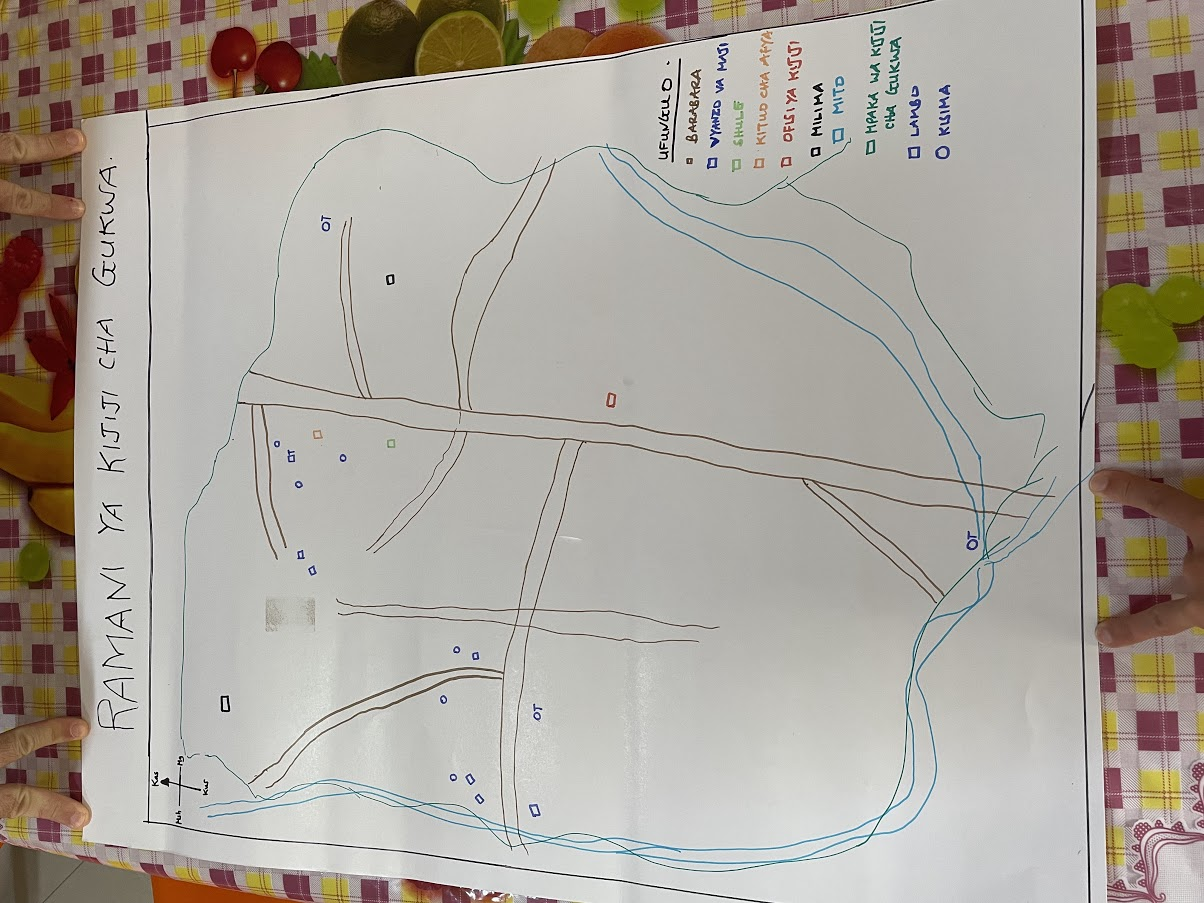** | **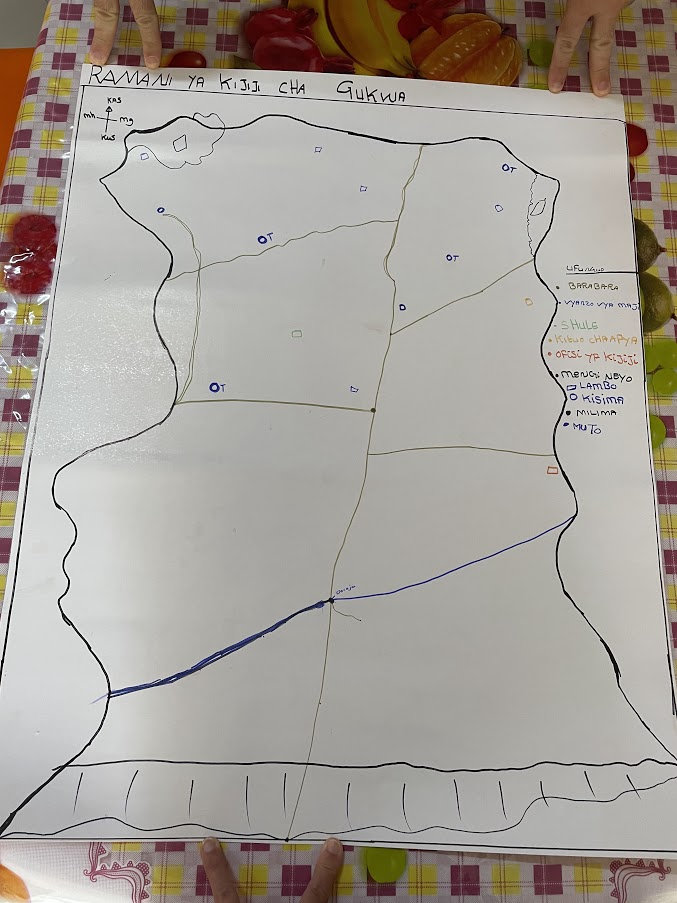** |
| **Mwagimagi, male** | **Mwagimagi, female** |
| **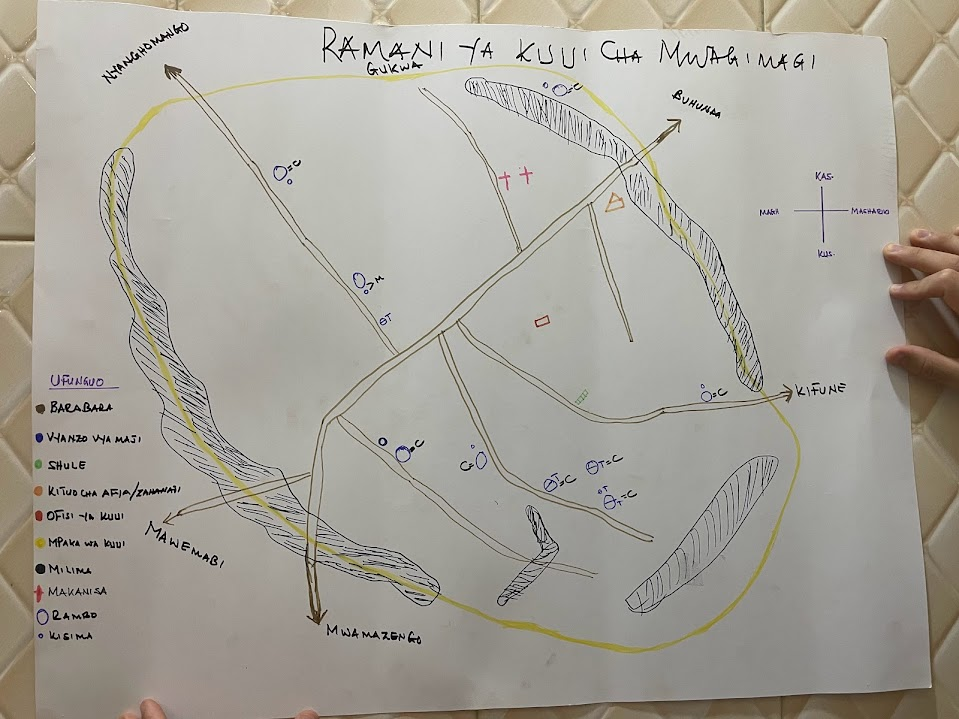** | **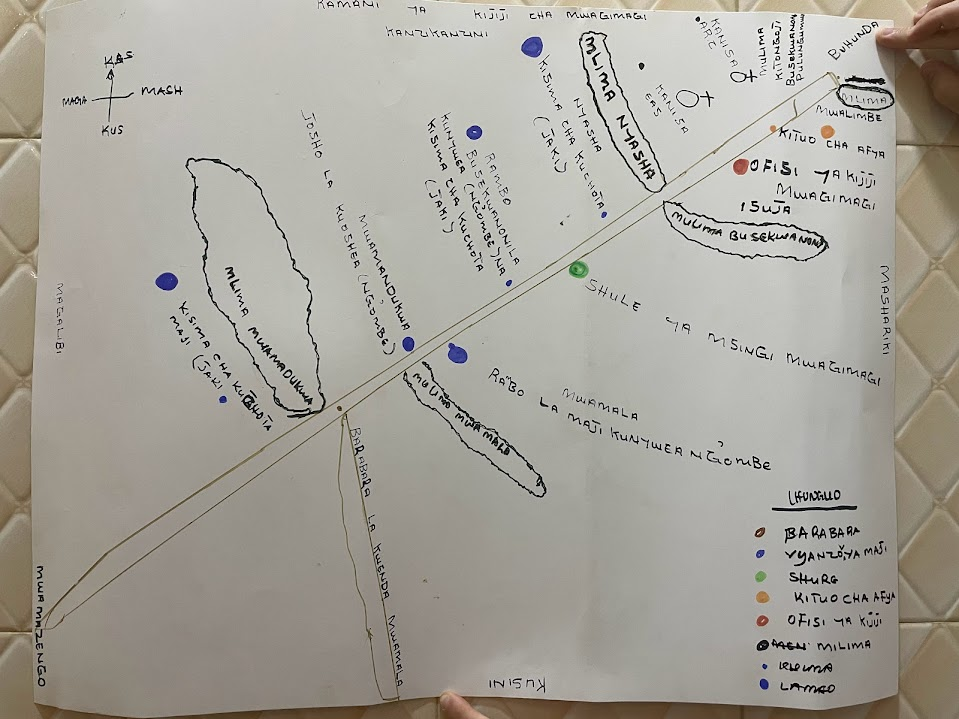** |
| **Isesa, male** | **Isesa, female** |
| **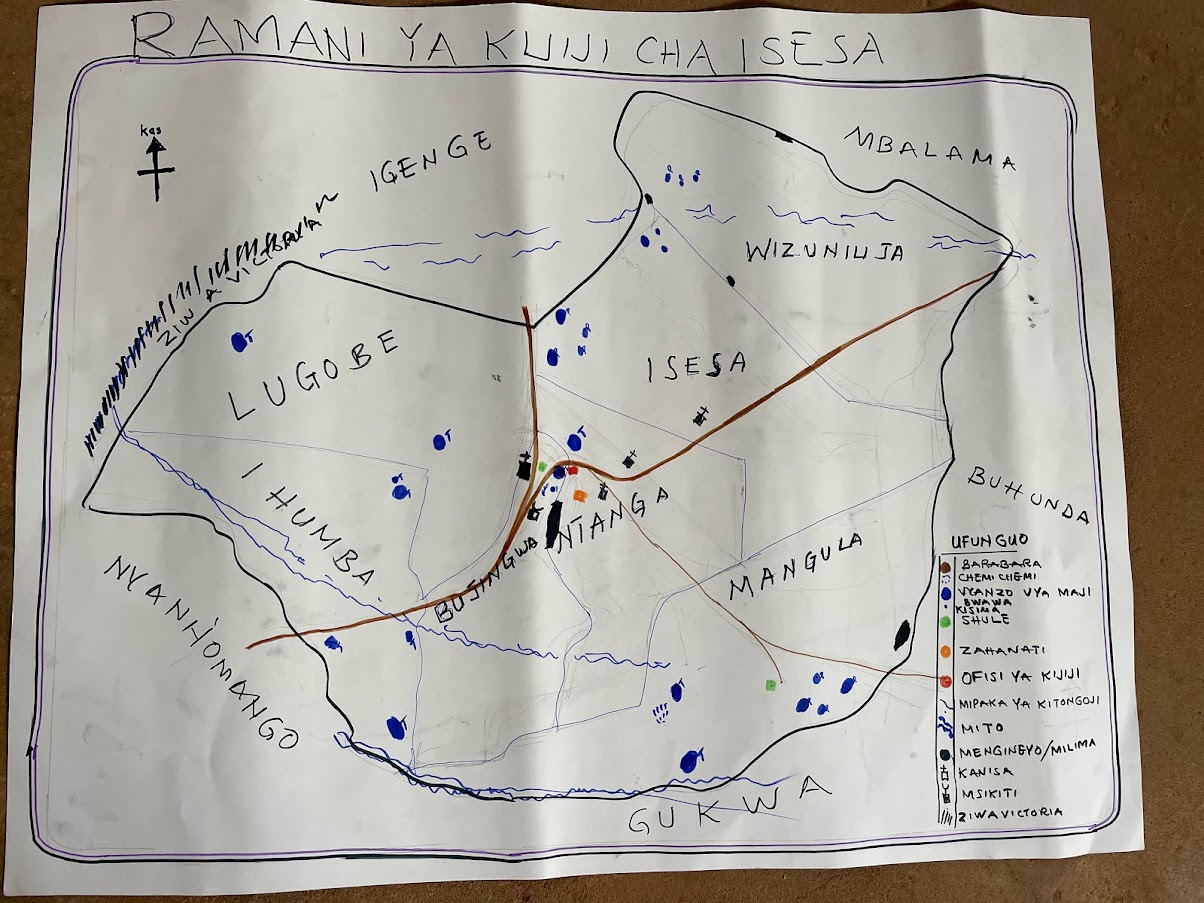** | **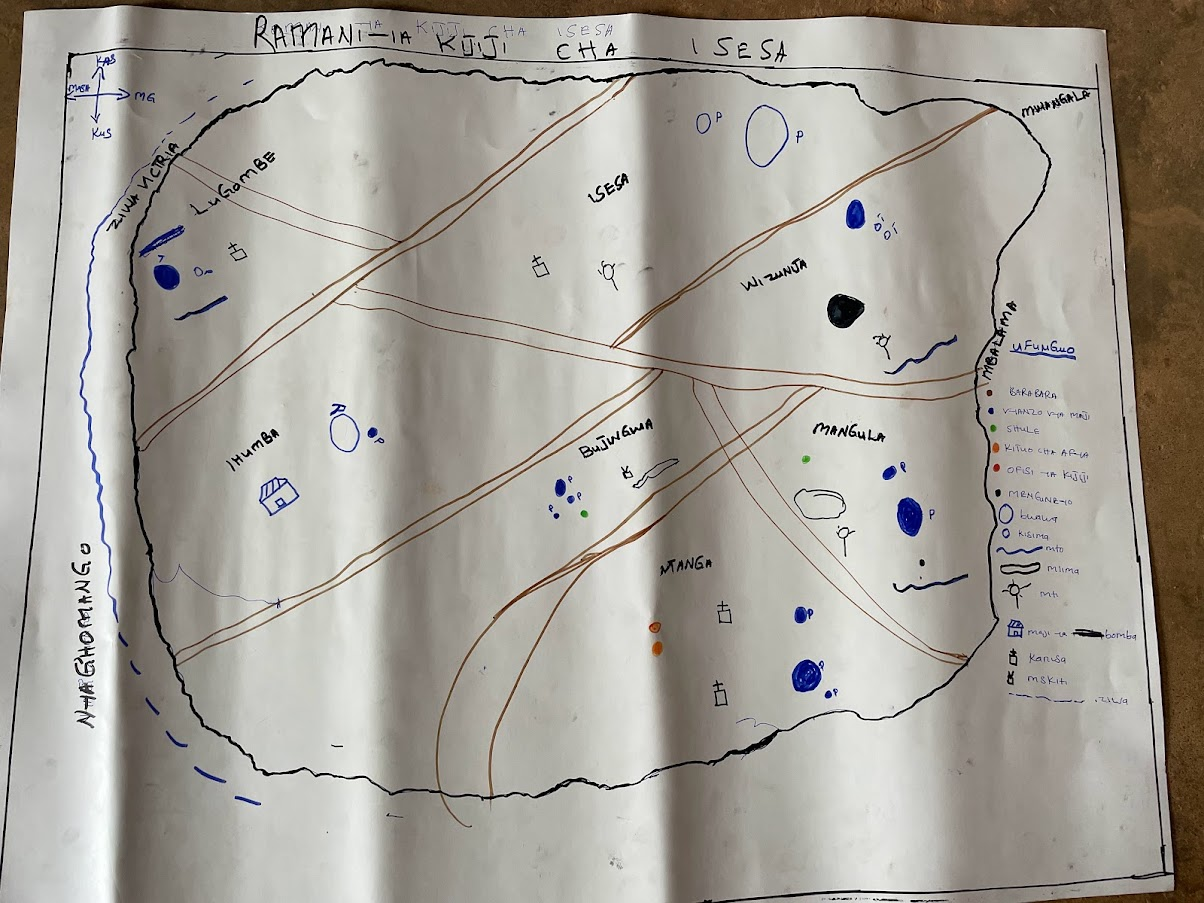** |
